# Supplementary figures and images for: Parafibromin governs cell polarity and centrosome assembly in Drosophila neural stem cells
Source: PLoS Biol. 2022 Oct 12;20(10):e3001834. doi: 10.1371/journal.pbio.3001834 (PMC9555638; doi:10.1371/journal.pbio.3001834)

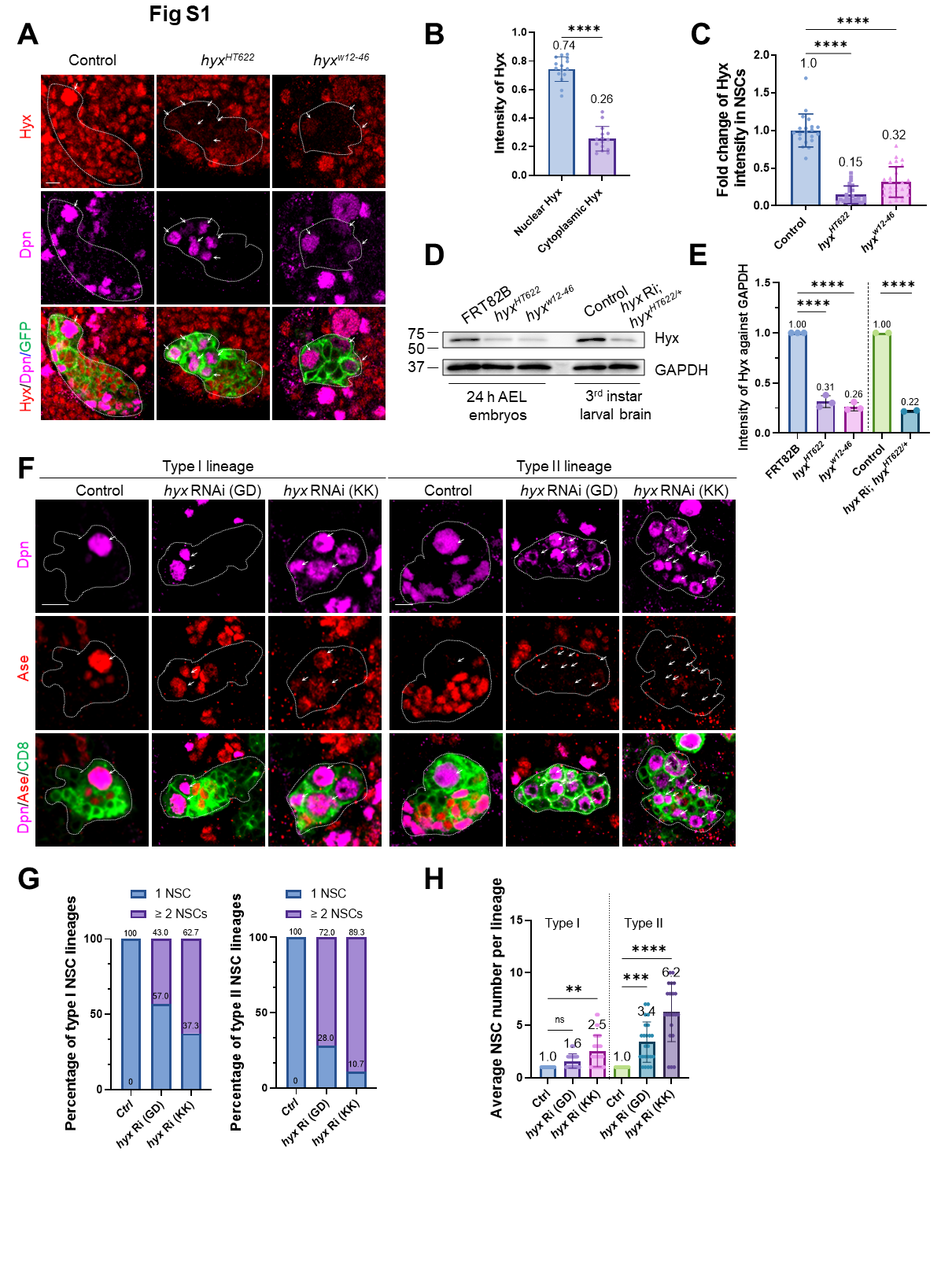

Supplement: S1 Fig — (A) MARCM clones of control (FRT82B; n = 20), hyxHT622 (n = 23), and hyxW12-46 (n = 25) were labelled for Hyx, Dpn, and GFP. (B) Immunofluorescence intensity (with SD) of both nuclear and cytoplasmic in wild-type NSCs. Nuclear Hyx: 0.74 ± 0.09-fold; cytoplasmic Hyx: 0.26 ± 0.09-fold, n = 15 NSC. (C) Fold change of immunofluorescence intensity (with SD) of Hyx in NSCs from control (FRT82B), hyxHT622, and hyxW12-46 (n = 40). Control: 1 ± 0.23-fold, n = 20; hyxHT622, 0.15 ± 0.18-fold, n = 25; hyxW12-46, 0.32 ± 0.20-fold, n = 28. (D) Western blotting analysis of 24 h AEL embryo extracts of control, hyxHT622, and hyxW12-46 as well as third instar larval brain extracts of control (UAS-β-gal Ri) and hyx Ri; hyxHT622/+ driven by insc-Gal4. Blots were probed with anti-Hyx antibody and anti-GAPDH antibody. A protein ladder was indicated on the left. (E) Fold change of Hyx protein levels normalizing against GAPDH (with SD) in D. Control (FRT82B): 1-fold; hyxHT622, 0.31 ± 0.06-fold; hyxW12-46, 0.26 ± 0.04-fold; control (UAS-β-gal Ri): 1-fold; hyx Ri; hyxHT622/+: 0.22 ± 0.007-fold. Minimum 2 biological replicates for all blots. (F) Type I and type II NSC lineages of control (UAS-β-Gal RNAi), hyx RNAi (GD/V28318), and hyx RNAi (KK/V103555) under the control of insc-Gal4 driver were labeled for Dpn, Ase, and CD8-GFP. (G) Percentage of NSC lineages with multiple NSCs (≥2 NSCs) for genotypes in F. Type I: control (UAS-β-Gal RNAi), 0, n = 57; hyx RNAi (GD), 43.0%, n = 92; hyx RNAi (KK), 62.7%, n = 73. Type II: control (UAS-β-Gal RNAi), 0, n = 64; hyx RNAi (GD), 72.0%, n = 83; hyx RNAi (KK), 89.3%, n = 56. (H) Average NSC number per NSC lineage (with SD) for genotypes in F. Type I: control (UAS-β-Gal RNAi), 1.0, n = 15; hyx RNAi (GD), 1.6 ± 0.68, n = 29; hyx RNAi (KK), 2.5 ± 1.50, n = 35. Type II: control (UAS-β-Gal RNAi), 1.0, n = 12; hyx RNAi (GD), 3.4 ± 1.92, n = 23; hyx RNAi (KK), 6.2 ± 2.8, n = 21. Statistical significances were determined by unpaired two-tailed Student t test in [file pbio.3001834.s001.TIF]

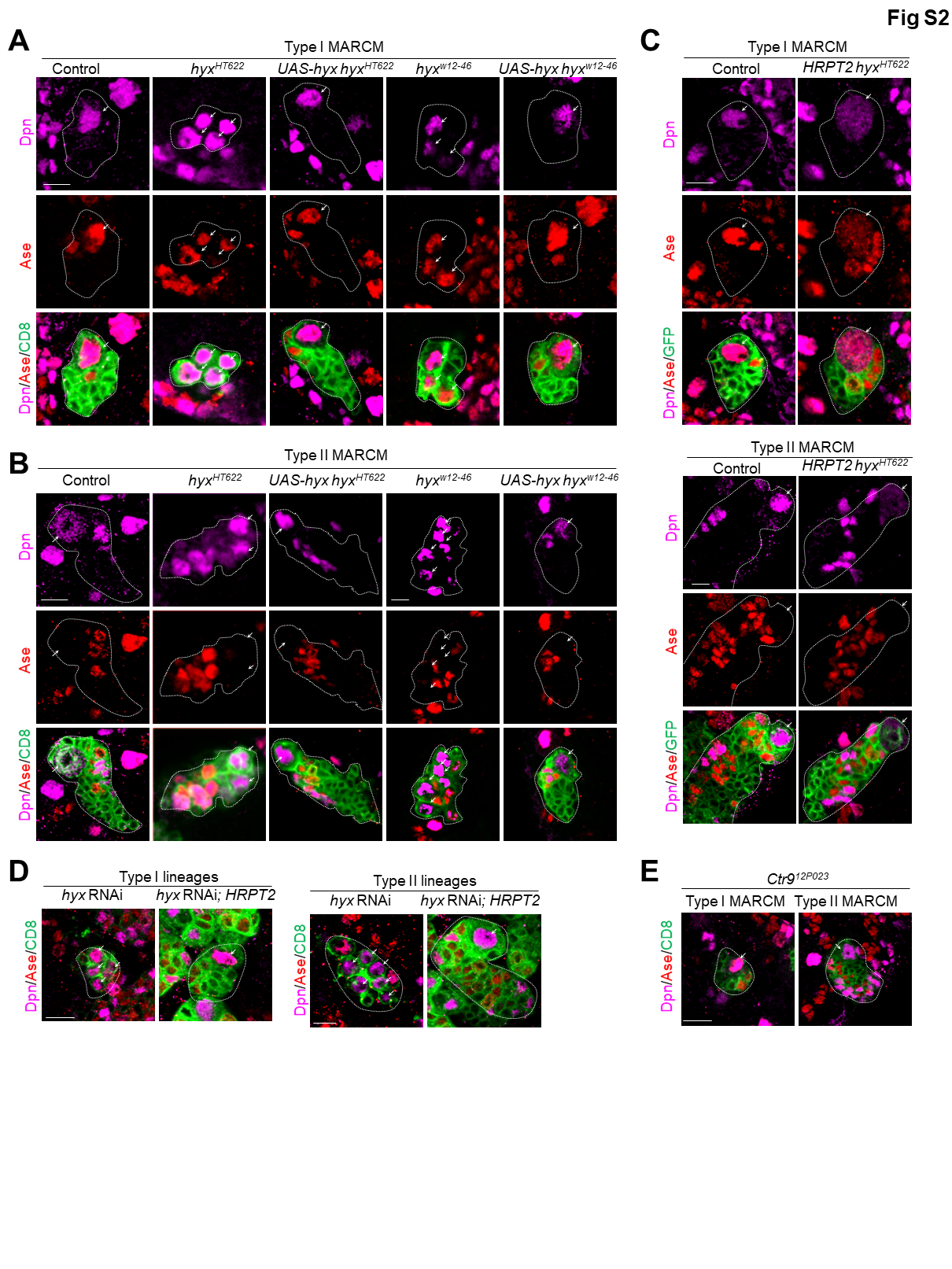

Supplement: S2 Fig — (A) Type I MARCM clones of control (FRT82B; n = 20), hyxHT622 (n = 17), hyxW12-46 (n = 30), UAS-hyx hyxHT622 (n = 21), and UAS-hyx hyxW12-46 (n = 40) were labelled for Dpn, Ase, and CD8. Ectopic NSCs were observed in 88.2% of hyxHT622 and 36.7% of hyxw12-46 larvae, but not in the control or rescued larvae. (B) Type II MARCM clones of control (FRT82B), hyxHT622, hyxW12-46, UAS-hyx hyxHT622, and UAS-hyx hyxW12-46 were labelled for Dpn, Ase, and CD8. Ectopic NSCs were observed in hyxHT622 (81.0%, n = 21) and hyxw12-46 (78.5%, n = 30) larvae, but not in control (n = 20), UAS-hyx hyxHT622 (n = 17) and UAS-hyx hyxW12-46 (n = 40) larvae. (C) MARCM clones of UAS-HRPT2 hyxHT622 type I (n = 30) and type II (n = 7) were labelled for Dpn, Ase, and GFP. (D) Type I and type II NSC lineages from hyx RNAi (V103555 with UAS-CD8-GFP) and UAS-HRPT2; hyx RNAi under the control of insc-Gal4 driver were labelled for Dpn, Ase, and CD8 (n = 10 brain lobes for each genotype). (E) Type I (n = 12) and type II (n = 16) MARCM clones from ctr912P023 were labelled for Dpn, Ase, and CD8. Clones/lineages are outlined by white-dotted lines. NSCs are indicated by white arrows. Scale bars: 5 μm. hyx, Hyrax; MARCM, mosaic analysis with a repressible cell marker; NSC, neural stem cell; RNAi, RNA interference. (TIF) [file pbio.3001834.s002.TIF]

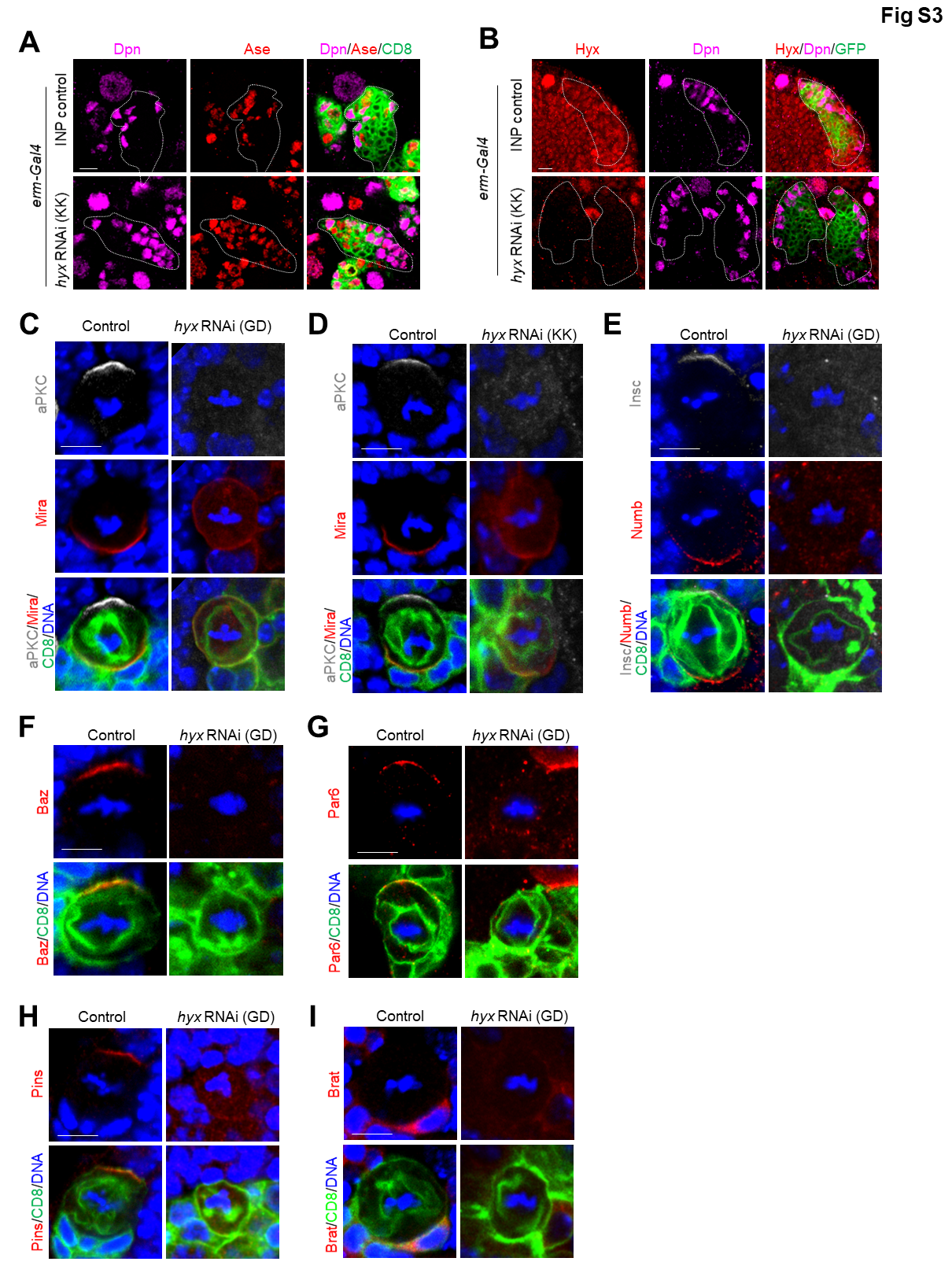

Supplement: S3 Fig — (A) INP lineages of control (UAS-Dicer2) and hyx RNAi (KK/V103555 with UAS-Dicer2) driven by erm-Gal4, UAS-CD8-GFP were labelled for Dpn, Ase, and CD8 (n = 30 for both). (B) INP lineages of control (UAS-Dicer2) and hyx RNAi (KK with UAS-Dicer2) driven by erm-Gal4, UAS-CD8-GFP were labelled for Hyx, Dpn, and CD8 (n = 30 and n = 45, respectively). (C) Metaphase NSCs of control (insc>CD8-GFP; n = 50) and hyx RNAi (GD/V28318 with UAS-CD8-GFP) type II lineages were labeled for aPKC, Mira, CD8, and DNA. hyx RNAi: aPKC delocalization, 100%, n = 50; Mira delocalization, 70%, n = 50. (D) Metaphase NSCs from control (insc>CD8-GFP) and hyx RNAi (KK/V103555 with UAS-CD8-GFP) type II lineages were labeled for aPKC, Mira, CD8, and DNA. In hyx RNAi, delocalization of aPKC: 100%; Mira: 90%; n = 50 for all. (E) Metaphase NSCs from control (insc>CD8-GFP) and hyx RNAi (GD/V28318 with UAS-CD8-GFP) type II lineages were labeled with Insc, Numb, CD8, and DNA. hyx RNAi, 100% delocalization of Insc and Numb; n = 50 for all. (F) Metaphase NSCs from control (insc>CD8-GFP) and hyx RNAi (GD/V28318 with UAS-CD8-GFP) type II lineages were labeled for Baz, CD8, and DNA; hyx RNAi: 100% delocalization of Baz; n = 50 for both genotypes. (G) Metaphase NSCs from control (insc>CD8-GFP) and hyx RNAi (GD/V28318 with UAS-CD8-GFP) type II lineages were labeled for Par6, CD8, and DNA. hyx RNAi: Par6 delocalization, 100%; n = 50 for both genotypes. (H) Metaphase NSCs from control (insc>CD8-GFP) and hyx RNAi (GD/V28318 with UAS-CD8-GFP) type II lineages were labeled for Pins, CD8, and DNA. hyx RNAi: Pins delocalization, 100%; n = 50 for both genotypes. (I) Metaphase NSCs from control (insc>CD8-GFP; n = 50) and hyx RNAi (GD/V28318 with UAS-CD8-GFP) type II lineages were labeled for Brat, CD8, and DNA. hyx RNAi: Brat delocalization, 100%; n = 50 for both genotypes. INP lineages are outlined by white-dotted lines. Scale bars: 5 μm. aPKC, atypical PKC; Baz, Bazooka; Hyx, Hyrax; Insc, Inscuteable; MARCM, mosaic an [file pbio.3001834.s003.TIF]

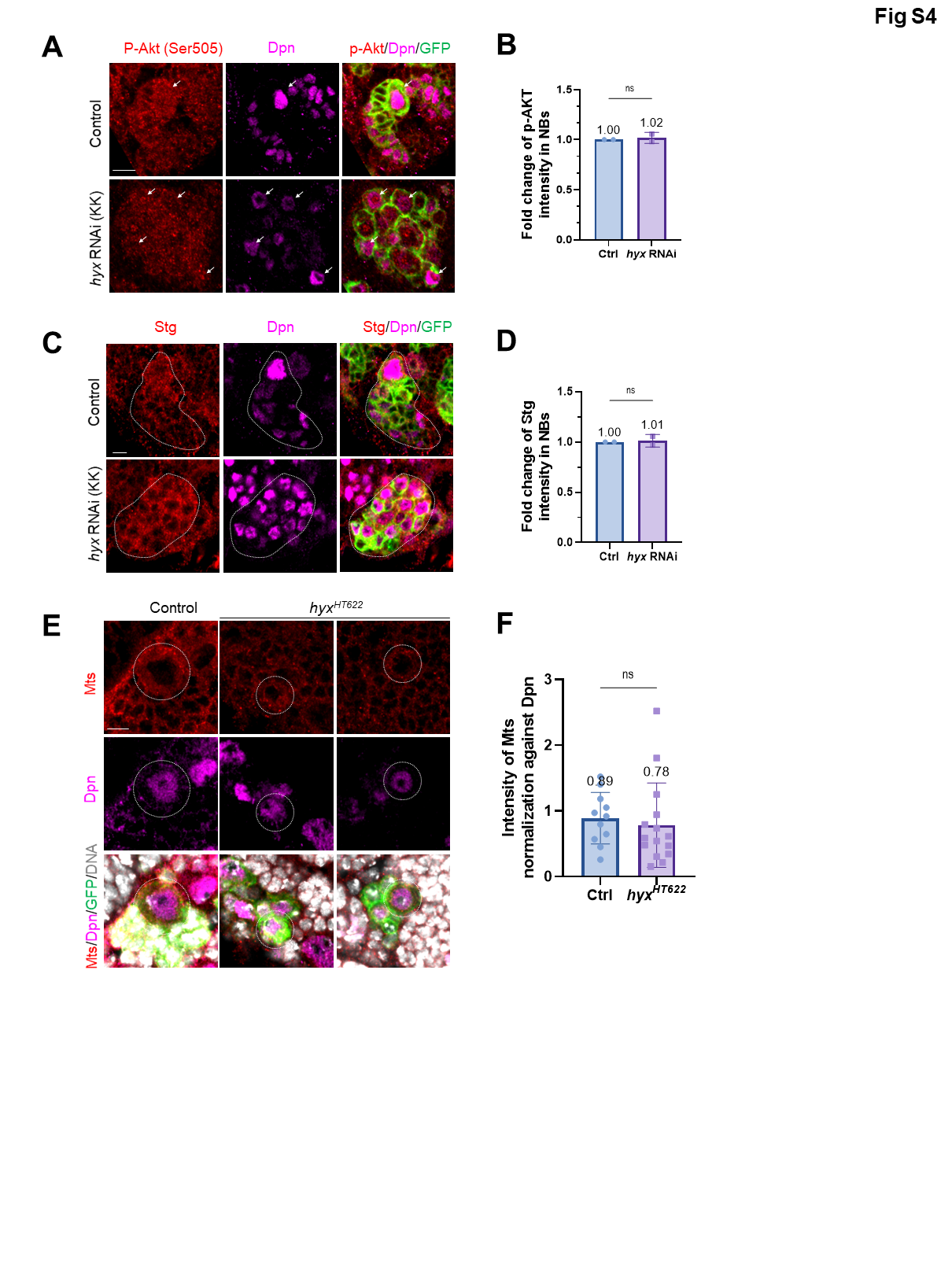

Supplement: S4 Fig — (A) NSC lineages from control (UAS-β-Gal RNAi, n = 54) and hyx RNAi (KK, n = 55) were labelled for P-Akt (Ser505), Dpn, and GFP. (B) Fold change of the immunofluorescence intensity of p-Akt (with SD) in NSCs for genotypes in A. Control: 1-fold; hyx RNAi: 1.02 ± 0.05-fold. (C) NSC lineages from control (UAS-β-Gal RNAi, n = 46) and hyx RNAi (KK, n = 65) were stained for Stg, Dpn, and GFP. (D) Fold change of the immunofluorescence intensity of Stg (with SD) in NSCs for genotypes in C. Control: 1-fold; hyx RNAi: 1.01 ± 0.06-fold. (E) NSCs from MARCM clones of control (FRT82B; n = 11) and hyxHT622 (n = 15) were probed with Mts, Dpn, GFP, and DNA. (F) Immunofluorescence intensity of Mts after normalization against Dpn (with SD) for genotypes in E. Control: 0.89 ± 0.39-fold; hyxHT622: 0.78 ± 0.64-fold. RNAi was controlled by insc-Gal4 in A and C. White arrows indicate NSCs in A. NSC lineages/NSCs are outlined by white-dotted lines in C and E. Statistical significances were determined by unpaired two-tailed Student t test in B, D, and F. ns = 0.6846 in B; ns = 0.8036 in D; ns = 0.6358 in F. Scale bars: 5 μm. The underlying data for this figure can be found in the S1 Data. Hyx, Hyrax; MARCM, mosaic analysis with a repressible cell marker; Mts, microtubule star; NSC, neural stem cell; PP2A, phosphatase 2A; RNAi, RNA interference; Stg, String. (TIF) [file pbio.3001834.s004.TIF]

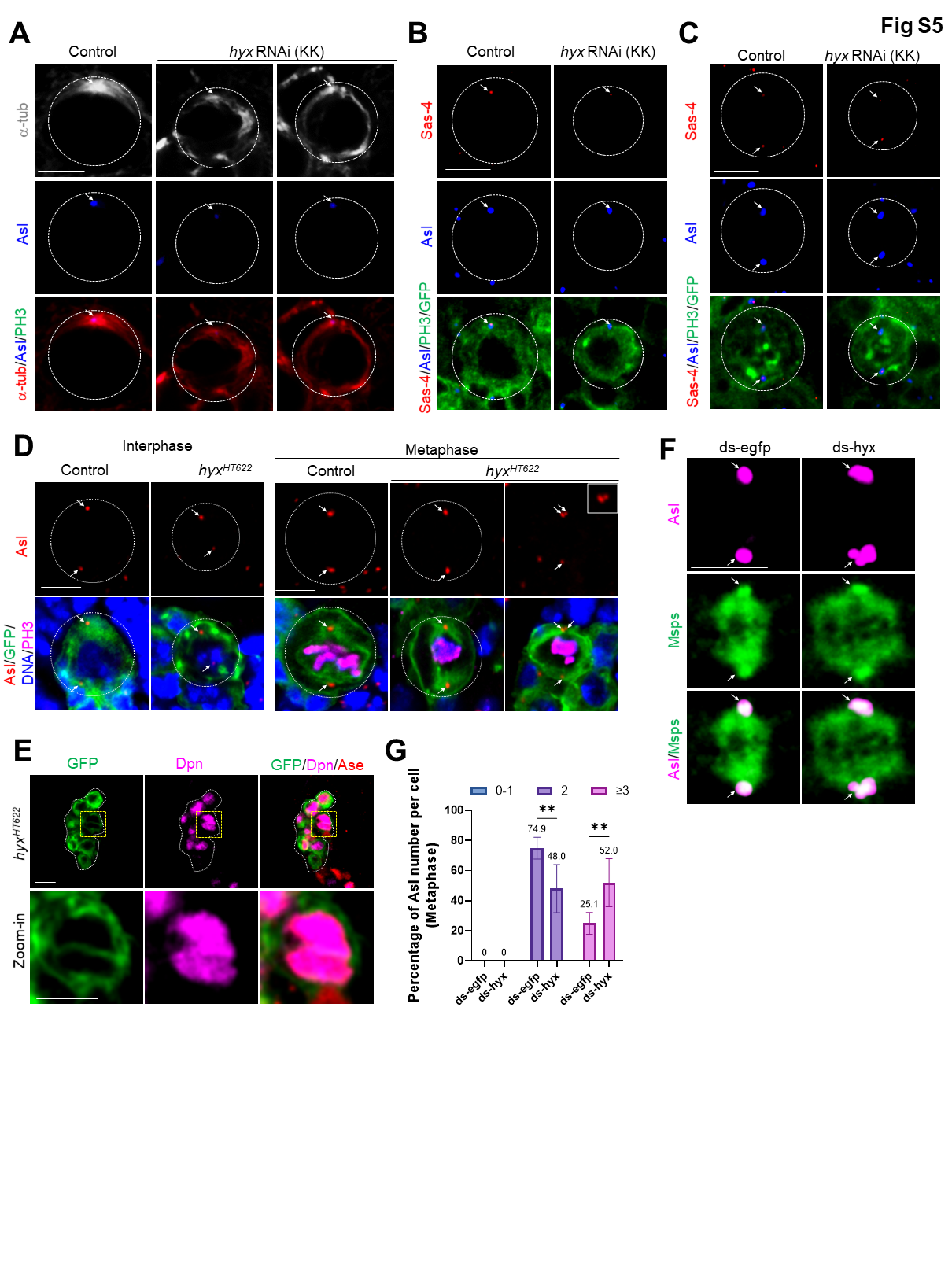

Supplement: S5 Fig — (A) Interphase NSCs from control (UAS-β-Gal RNAi; 100% aster formation, n = 20) and hyx RNAi (KK/V103555; n = 22) were labelled for α-tub, Asl, and PH3. (B) Interphase NSCs of control (UAS-β-Gal RNAi; n = 23) and hyx RNAi (KK/V103555; n = 20) were labelled for Sas-4, Asl, and PH3. (C) Prometa/metaphase NSCs of control (UAS-β-Gal RNAi) and hyx RNAi (KK/V103555) were labeled for Sas-4, Asl, and PH3 (n = 23 for both). (D) Interphase and metaphase NSCs from control and hyxHT622 MARCM clones were probed with Asl, GFP, DNA, and PH3. Centrioles marked by Asl are pointed out by arrows. Control interphase (n = 21) and metaphase NSCs (n = 20) typically contain 2 Asl-positive centrioles. Two centrioles marked by Asl were always seen in hyxHT622 interphase NSCs (n = 20); multiple centrioles labelled by Asl were observed in 28.1% (n = 32) metaphase NSCs from hyxHT622 MARCM clones and the rest of metaphase (79.1%) NSCs showing 2 Asl-positive centrioles. NSCs/NSC lineages are outlined and Zoom-in areas are boxed. (E) hyxHT622 MARCM clones were labelled with GFP, Dpn, and Ase. Cytokinesis delay was shown (n = 23). (F) Metaphase ds-egfp-treated S2 cells (n = 195) and ds-hyx-treated S2 cells (n = 172) were labeled for Msps and Asl. (G) Quantification graph displaying the percentage of metaphase S2 cells with the indicated number of Asl per NSC in G. Percentage of metaphase S2s with multiple Asl (≥3): ds-egfp, 52.0 ± 7.3%; ds-egfp, 25.1 ± 15.9%. hyx knockdown was driven by insc-Gal4 in A-C. Arrows indicate the centrosomes. Scale bars: 5 μm. The underlying data for this figure can be found in the S1 Data. Asl, Asterless; Hyx, Hyrax; MARCM, mosaic analysis with a repressible cell marker; Msps, Mini spindles; NSC, neural stem cell; RNAi, RNA interference; Sas-4, Spindle assembly abnormal 4. (TIF) [file pbio.3001834.s005.tif]

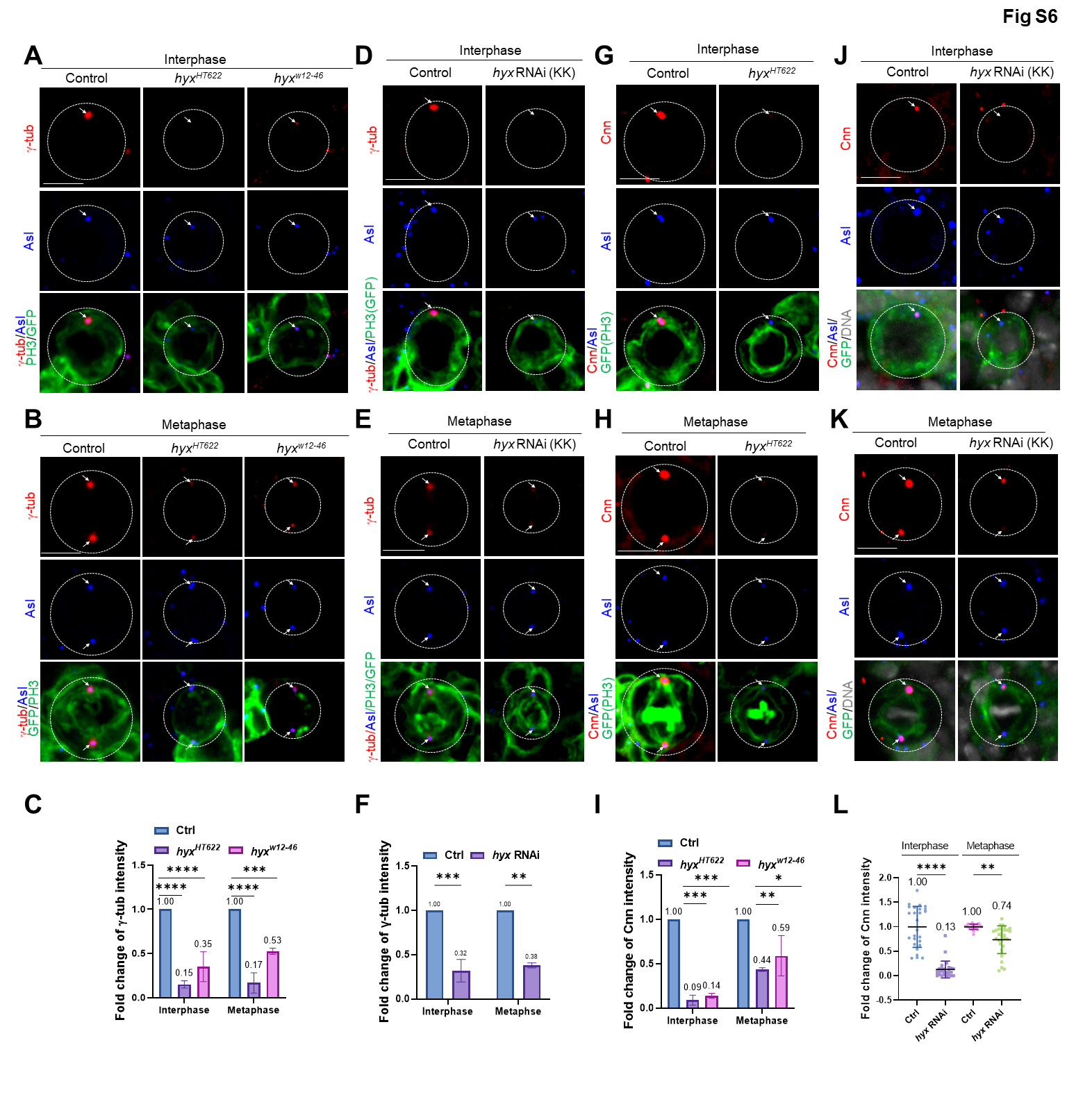

Supplement: S6 Fig — (A) Interphase NSCs of MARCM clones in control (FRT82B) and hyxHT622 were labelled for γ-tub, Asl, GFP, and PH3. γ-tub delocalization: control, 0%, n = 26; hyxHT622, 95.7%, n = 23; hyxw12-46, 54.8%, n = 42. (B) Metaphase NSCs of MARCM clones in control (FRT82B) and hyxHT622 were labelled for γ-tub, Asl, GFP, and PH3. γ-tub delocalization: control, 0%, n = 29; hyxHT622, 93.1%, n = 29; hyxw12-46, 70.8%, n = 24. (C) Quantification graph of the fold change of γ-tub intensity (with SD) in NSCs from A and B. Interphase: control, 1-fold; hyxHT622, 0.15 ± 0.04-fold; hyxw12-46, 0.35 ± 0.17-fold. Metaphase: control, 1-fold; hyxHT622, 0.17 ± 0.12-fold; hyxw12-46, 0.53 ± 0.04-fold. (D) Control (UAS-β-Gal RNAi; n = 36) and hyx RNAi (KK/V103555; n = 42) interphase NSCs were labelled for γ-tub, Asl, GFP, and PH3. (E) Control (UAS-β-Gal RNAi; n = 25) and hyx RNAi (KK/V103555; n = 32) metaphase NSCs were labelled for γ-tub, Asl, GFP, and PH3. In control, robust distribution of γ-tub was seen in 88.9% of interphase (A) and 92.0% of metaphase (B) NSCs. (F) Quantification graph of the fold change of γ-tub intensity (with SD) in NSCs from D and E. Interphase: control, 1-fold, n = 36; hyx RNAi, 0.32 ± 0.13-fold, n = 42. Metaphase: control, 1-fold, n = 25; hyx RNAi, 0.38 ± 0.03-fold, n = 32. (G) Interphase NSCs of control (FRT82B; n = 51) and hyxHT622 (n = 33) MARCM clones were labelled for Cnn, Asl, GFP, and PH3. (H) Metaphase MARCM clones of control (FRT82B; all NSCs have robust Cnn localization, n = 27) and hyxHT622 (n = 29) were labelled for Cnn, Asl, GFP, and PH3. (I) Quantification graph of the fold change of Cnn intensity (with SD) in NSCs from G and H. Interphase: control, 1-fold, n = 51; hyxHT622, 0.09 ± 0.06-fold, n = 33; hyxw12-46, 0.14 ± 0.03-fold, n = 12. Metaphase: control, 1-fold, n = 27; hyxHT622, 0.44 ± 0.02-fold, n = 29; hyxw12-46, 0.59 ± 0.23-fold, n = 18. (J) Interphase NSCs from control (UAS-β-Gal RNAi; 96.3% robust Cnn signal) and hyx RNAi (KK/V103555) were labelled [file pbio.3001834.s006.TIF]

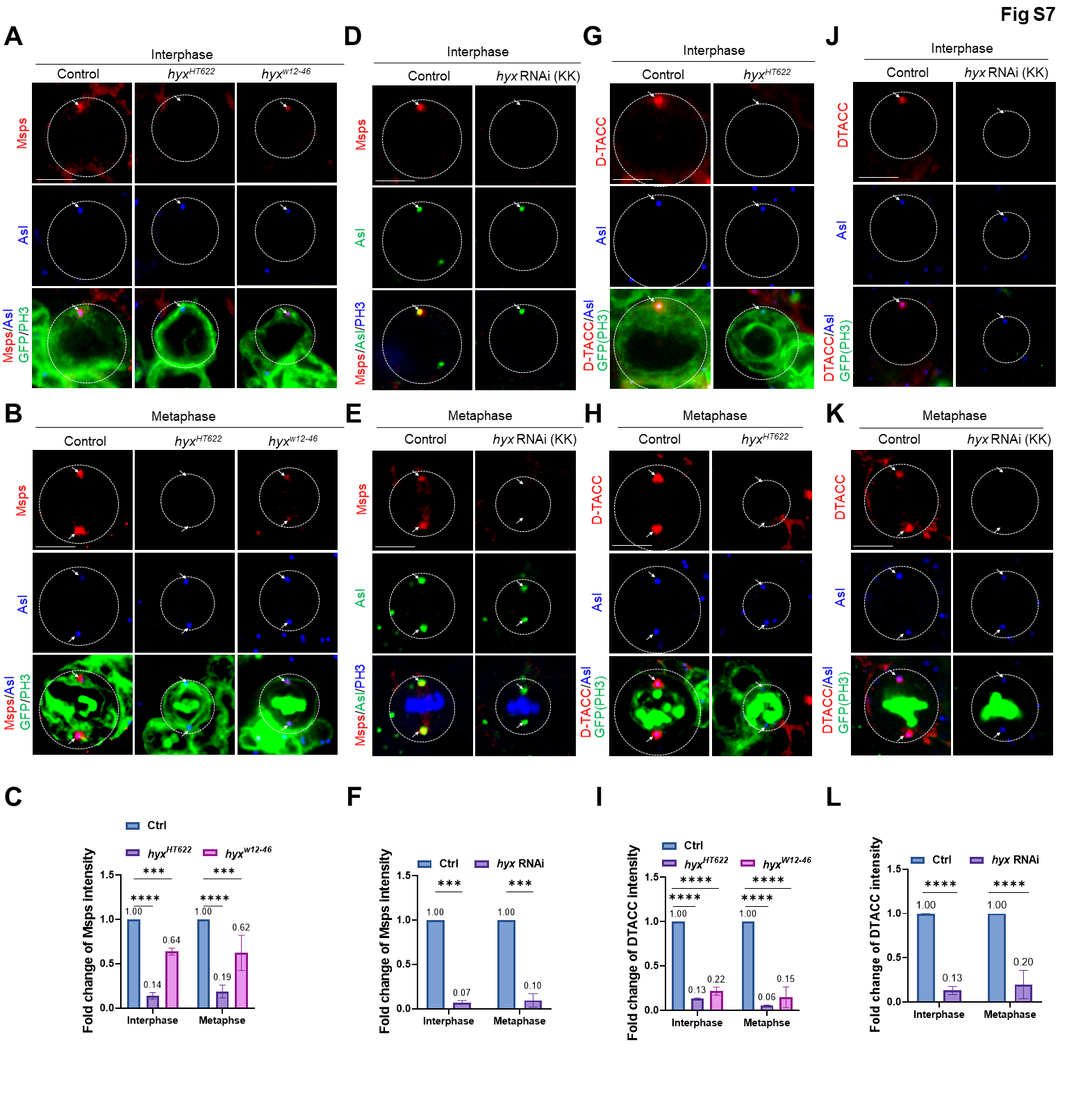

Supplement: S7 Fig — (A) Interphase NSCs of MARCM clones in control (FRT82B; 98.1% of NSCs have strong Msps localization at the centrosomes) and hyxHT622 were analyzed by Msps, Asl, GFP, and PH3. Control, n = 54; hyxHT622, n = 44; hyxw12-46, n = 17. (B) Metaphase NSCs of control (FRT82B; all NSCs have Msps localization at the centrosomes, n = 32), hyxHT622 (n = 24), and hyxw12-46 (n = 15) MARCM clones were labelled for Msps, Asl, GFP, and PH3. (C) Quantification graph of the fold change of Msps intensity (with SD) in NSCs from A and B. Interphase: control, 1-fold, n = 54; hyxHT622, 0.14 ± 0.04-fold, n = 44; hyxw12-46, 0.64 ± 0.04-fold, n = 17. Metaphase: control, 1-fold, n = 40; hyxHT622, 0.19 ± 0.07-fold, n = 24; hyxw12-46, 0.62 ± 0.20-fold, n = 15. (D) Interphase NSCs of control (UAS-β-Gal RNAi) and hyx RNAi (KK/V103555) were labelled for Msps, Asl, and PH3. Msps delocalization at the centrosomes: control: 0%, n = 55; hyx RNAi, 82.5%, n = 57. (E) Metaphase NSCs of control (UAS-β-Gal RNAi) and hyx RNAi (KK/V103555) were labelled for Msps, Asl, and PH3. Msps localization at the centrosomes: control: 98.5%, n = 55; hyx RNAi, 16.4%, n = 67. (F) Quantification graph of the fold change of Msps intensity (with SD) in NSCs from D and E. Interphase: control, 1-fold, n = 25; hyx RNAi, 0.07 ± 0.03-fold, n = 25. Metaphase: control, 1-fold, n = 25; hyx RNAi, 0.10 ± 0.11-fold, n = 26. (G) Interphase NSCs from control (FRT82B; n = 41) and hyxHT622 (n = 25) MARCM clones were labelled for DTACC, Asl, GFP, and PH3. (H) MARCM clones of control (FRT82B; 100% D-TACC localization, n = 18) and hyxHT622 (n = 21) were labelled for DTACC, Asl, GFP, and PH3. (I) Quantification graph of the fold change of DTACC intensity (with SD) in NSCs from G and H. Interphase: control, 1-fold, n = 21; hyxHT622, 0.13 ± 0.01-fold, n = 25; hyxw12-46, 0.22 ± 0.06-fold, n = 22. Metaphase: control, 1-fold, n = 18; hyxHT622, 0.06 ± 0.007, n = 21; hyxw12-46, 0.15- ± 0.17-fold, n = 15. (J) Interphase NSCs of control (UAS-β-Gal RNAi) [file pbio.3001834.s007.TIF]

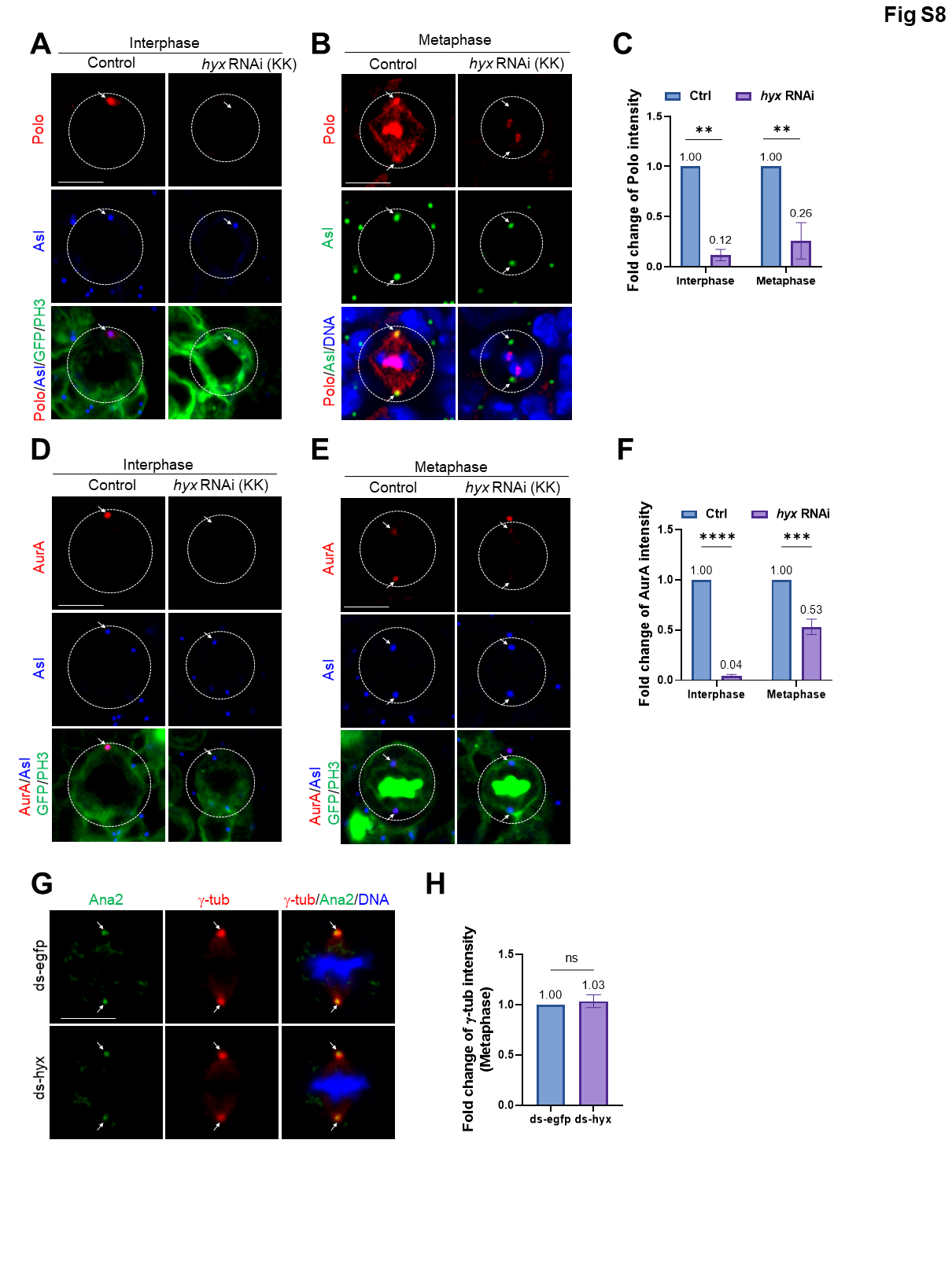

Supplement: S8 Fig — (A) Interphase NSCs of control (UAS-β-Gal RNAi; n = 29) and hyx RNAi (KK/V103555; n = 26) were labelled with Polo, Asl, and PH3. (B) Metaphase NSCs of control (UAS-β-Gal RNAi; Polo present at the centrosomes in 95.0% of NSCs, n = 20) and hyx RNAi (KK/V103555; n = 23) were labelled for Polo, Asl, and DNA. (C) Quantification graph showing the fold change of Polo intensity (with SD) in A and B. Interphase: control, 1-fold, n = 29; hyx RNAi, 0.12 ± 0.06-fold, n = 26. Metaphase: control, 1-fold, n = 20; hyx RNAi, 0.26 ± 0.18-fold, n = 24. (D) Interphase NSCs of control (UAS-β-Gal RNAi; AurA present at the centrosomes in 96.6% of NSCs, n = 29) and hyx RNAi (KK/V103555; n = 24) were labelled for AurA, Asl, GFP, and PH3. (E) Metaphase NSCs of control (UAS-β-Gal RNAi; AurA observed at the centrosomes in 96.4% of NSCs, n = 28) and hyx RNAi (KK/V103555; n = 30) were labelled for AurA, Asl, GFP, and PH3. Polo and AurA are properly localized in all control NSCs in A-E. (F) Quantification graph showing the fold change of AurA intensity (with SD) in D and E. Interphase: control, 1-fold, n = 29; hyx RNAi, 0.04 ± 0.02-fold, n = 24. Metaphase: control, 1-fold, n = 28; hyx RNAi, 0.53 ± 0.08-fold, n = 30. (G) Metaphase cells from ds-egfp-treated S2 cells and ds-hyx-treated S2 cells were labelled for Ana2, γ-tub, and DNA. (H) Quantification graph showing the fold change of γ-tub intensity in G. ds-egfp, 1-fold, n = 69; ds-hyx, 1.03 ± 0.06-fold, n = 52. hyx knockdown was under the control of insc-Gal4 in A-F. NSCs are outlined by white-dotted lines. Centrosomes are pointed by arrows. Statistical significances were determined by two-way ANOVA with multiple comparison in C and F. Unpaired two-tailed Student t test was performed in H. In C, **p = 0.0015 for interphase, **p = 0.0029 for metaphase; in F, ****p < 0.0001, ***p = 0.0006; in H, ns = 0.4023. Scale bars: 5 μm. The underlying data for this figure can be found in the S1 Data. Ana2, Anastral spindle 2; Asl, Asterless; AurA, Aurora-A; [file pbio.3001834.s008.TIF]

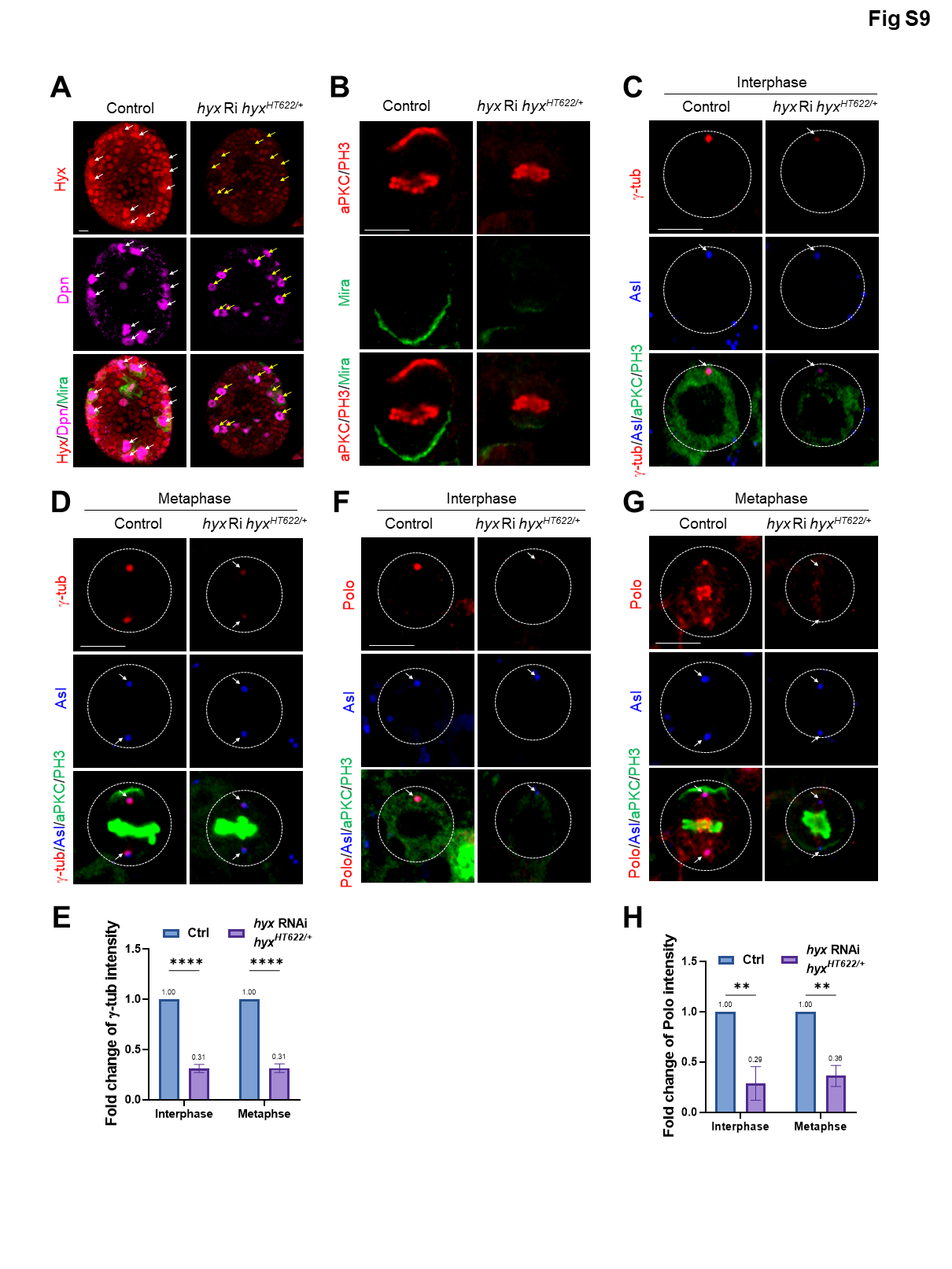

Supplement: S9 Fig — (A) 24 h ALH brains from control (UAS-β-Gal RNAi; n = 10 brain lobes) and hyx RNAi hyxHT622 (KK/V103555, n = 10 brain lobes) were labelled with Hyx, Dpn, and Mira. Arrows indicate NSCs. (B) 24 h ALH brains from control (UAS-β-Gal RNAi; both aPKC and Mira formed crescent in all metaphase NSCs, n = 47) and hyx RNAi hyxHT622/+ (aPKC and Mira delocalized in 96.8% and 91.9% of NSCs, n = 62 for both) were examined by aPKC, Mira, and PH3. (C) At 24 h ALH, interphase NSCs of control (UAS-β-Gal RNAi; γ-tub present at the centrosomes in all NSCs examined, n = 34) and hyx RNAi hyxHT622 (KK/V103555; γ-tub delocalization at the centrosomes, 82.4%, n = 34) were labelled with γ-tub, Asl, aPKC, and PH3. (D) At 24 h ALH, metaphase NSCs of control (UAS-β-Gal RNAi; γ-tub present at the centrosomes in all NSCs observed, n = 34) and hyx RNAi hyxHT622 (KK/V103555; γ-tub delocalization at the centrosomes, 85%, n = 40) were labelled for γ-tub, Asl, aPKC, and PH3. (E) Quantification graph showing the fold change of γ-tub intensity (with SD) in C and D. Interphase: control, 1-fold, n = 34; hyx RNAi hyxHT622/+, 0.31 ± 0.04-fold, n = 34. Metaphase: control, 1-fold, n = 34; hyx RNAi hyxHT622/+, 0.31 ± 0.04-fold, n = 40. (F) Interphase NSCs of 24 h ALH control (UAS-β-Gal RNAi; Polo present at the centrosomes in 96.4% of NSCs, n = 28) and hyx RNAi hyxHT622 (KK/V103555; Polo delocalization at the centrosomes, 81.8%, n = 22) were labelled for Polo, Asl, aPKC, and PH3. (G) Metaphase NSCs of 24 h ALH control (UAS-β-Gal RNAi; Polo observed at the centrosomes in 95.5% of NSCs, n = 52) and hyx RNAi hyxHT622/+ (KK/V103555; Polo mislocalization at the centrosomes, 71.9%, n = 32) were labelled for Polo, Asl, aPKC, and PH3. (H) Quantification graph showing the fold change of Polo intensity (with SD) in F and G. Interphase: control, 1-fold, n = 28; hyx RNAi hyxHT622/+, 0.29 ± 0.17-fold, n = 22. Metaphase: control, 1-fold, n = 52; hyx RNAi hyxHT622/+, 0.36 ± 0.10-fold, n = 32. NSCs are outlined by white-dotte [file pbio.3001834.s009.TIF]

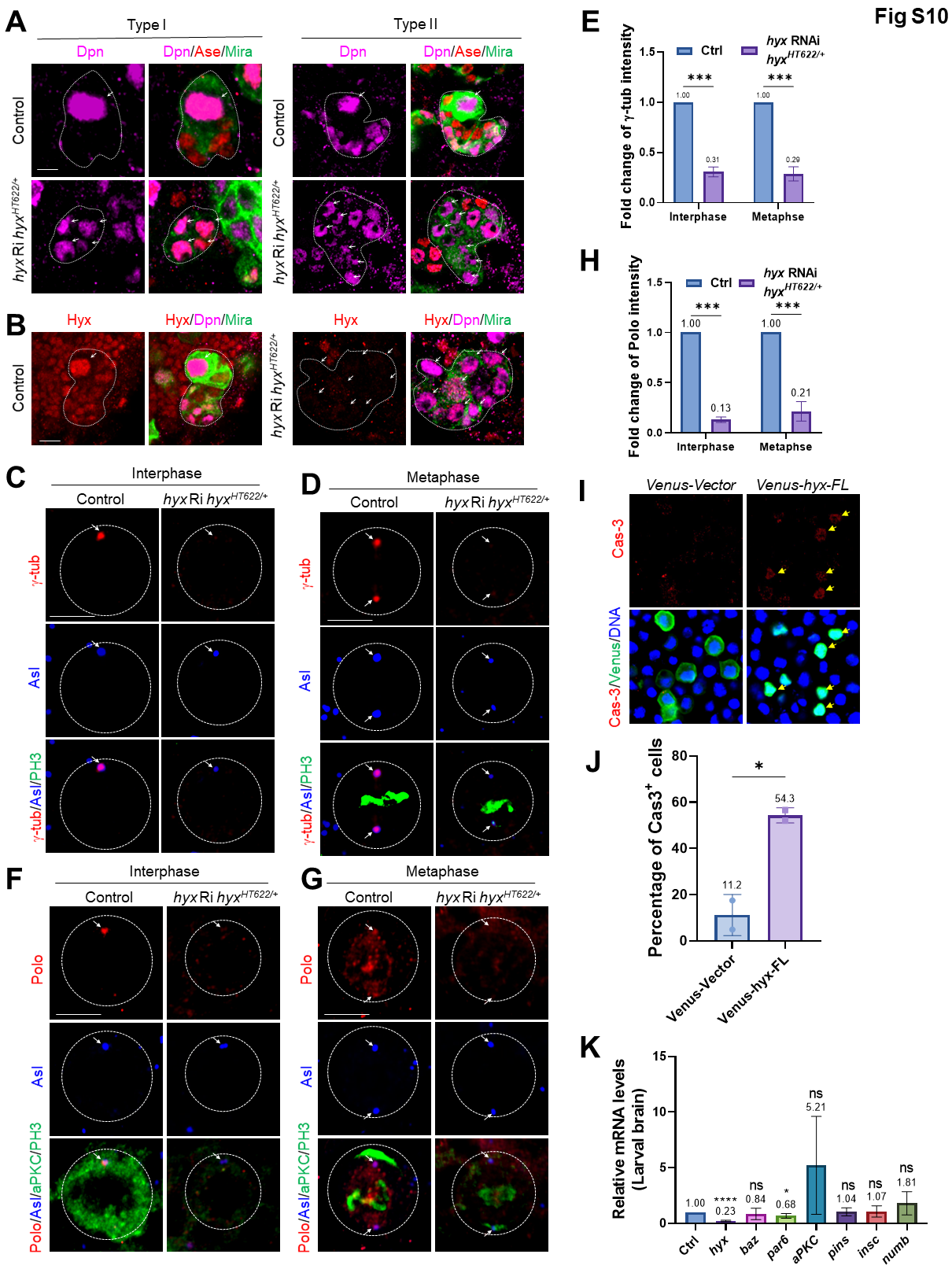

Supplement: S10 Fig — (A) Type I and type II NSC lineages from control (UAS-β-Gal RNAi; a single NSC was displayed in all lineages, type I, n = 20 and type II, n = 20) and hyx RNAi hyxHT622/+ (KK/V103555; ectopic NSCs were observed, type I, 84.3%, n = 51 and type II, 93.3%, n = 30) were labelled with Dpn, Ase, and Mira. (B) NSC lineages from control (UAS-β-Gal RNAi; n = 50) and hyx RNAi hyxHT622/+ (KK/V103555; Hyx protein levels were reduced in the nucleus of NSCs, 89.1%, n = 44) were examined with Hyx, Dpn, and Mira. (C) Interphase NSCs of control (UAS-β-Gal RNAi; γ-tub present at the centrosomes in all NSCs, n = 24) and hyx RNAi hyxHT622/+ (KK/V103555; Delocalization of γ-tub at the centrosomes in 88.9% of NSCs, n = 19) were labelled with γ-tub, Asl, and PH3. (D) Metaphase NSCs of control (UAS-β-Gal RNAi; γ-tub present at the centrosome in all NSCs, n = 25) and hyx RNAi hyxHT622/+ (KK/V103555; reduction of γ-tub protein levels at the centrosomes in 88.9% of NSCs, n = 18) were labelled for γ-tub, Asl, and PH3. (E) Quantification graph showing the fold change of γ-tub intensity (with SD) in C and D. Interphase: control, 1-fold, n = 24; hyx RNAi hyxHT622/+, 0.31 ± 0.05-fold, n = 19. Metaphase: control, 1-fold, n = 25; hyx RNAi hyxHT622/+, 0.29 ± 0.07-fold, n = 18. (F) Interphase NSCs of control (UAS-β-Gal RNAi; Polo present at the centrosomes in all NSCs examined, n = 27) and hyx RNAi hyxHT622/+ (KK/V103555; Polo delocalized at the centrosome in 84.6% of NSCs, n = 39) were labelled for Polo, Asl, aPKC, and PH3. (G) Metaphase NSCs of control (UAS-β-Gal RNAi; Polo observed at the centrosomes in all NSCs, n = 27) and hyx RNAi hyxHT622/+ (KK/V103555; Polo reduced at the centrosomes in 90.0% of NSCs, n = 20) were labelled for Polo, Asl, aPKC, and PH3. (H) Quantification graph showing the fold change of Polo intensity (with SD) in F and G. Interphase: control, 1-fold, n = 27; hyx RNAi hyxHT622/+, 0.13 ± 0.03-fold, n = 39. Metaphase: control, 1-fold, n = 27; hyx RNAi hyxHT622/+, 0.21 ± 0.10-fold [file pbio.3001834.s010.TIF]
