# Supplementary material for: Parafibromin governs cell polarity and centrosome assembly in Drosophila neural stem cells
Source: PLoS Biol. 2022 Oct 12;20(10):e3001834. doi: 10.1371/journal.pbio.3001834 (PMC9555638; doi:10.1371/journal.pbio.3001834)

Original uncropped Western blot for S1D Fig

Unlabeled Labeled

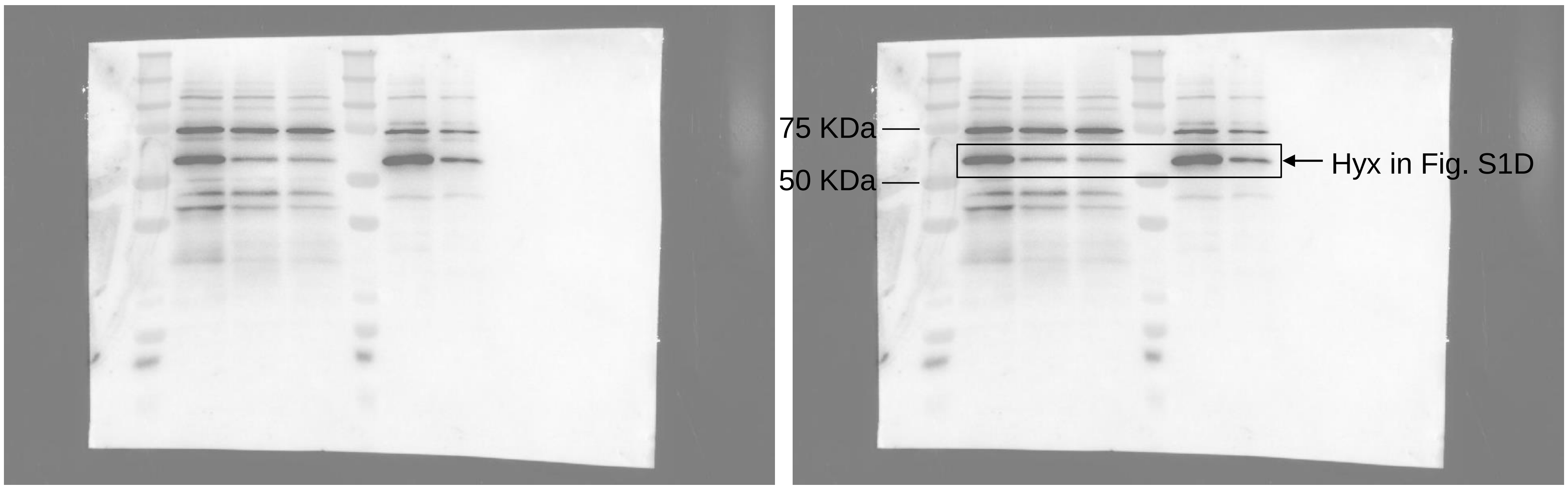

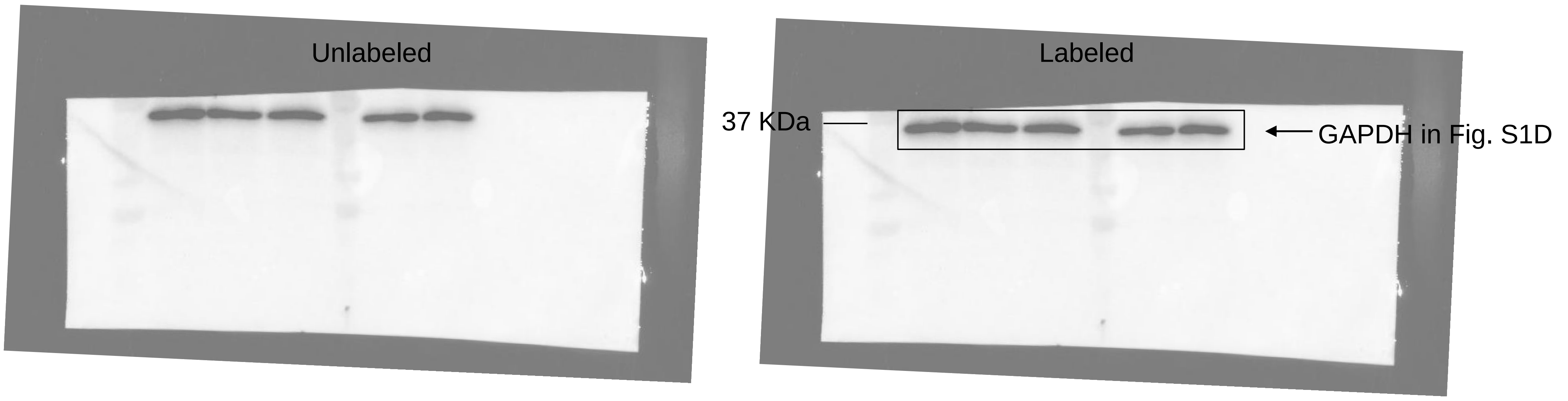

Original uncropped Western blot for Fig 6P

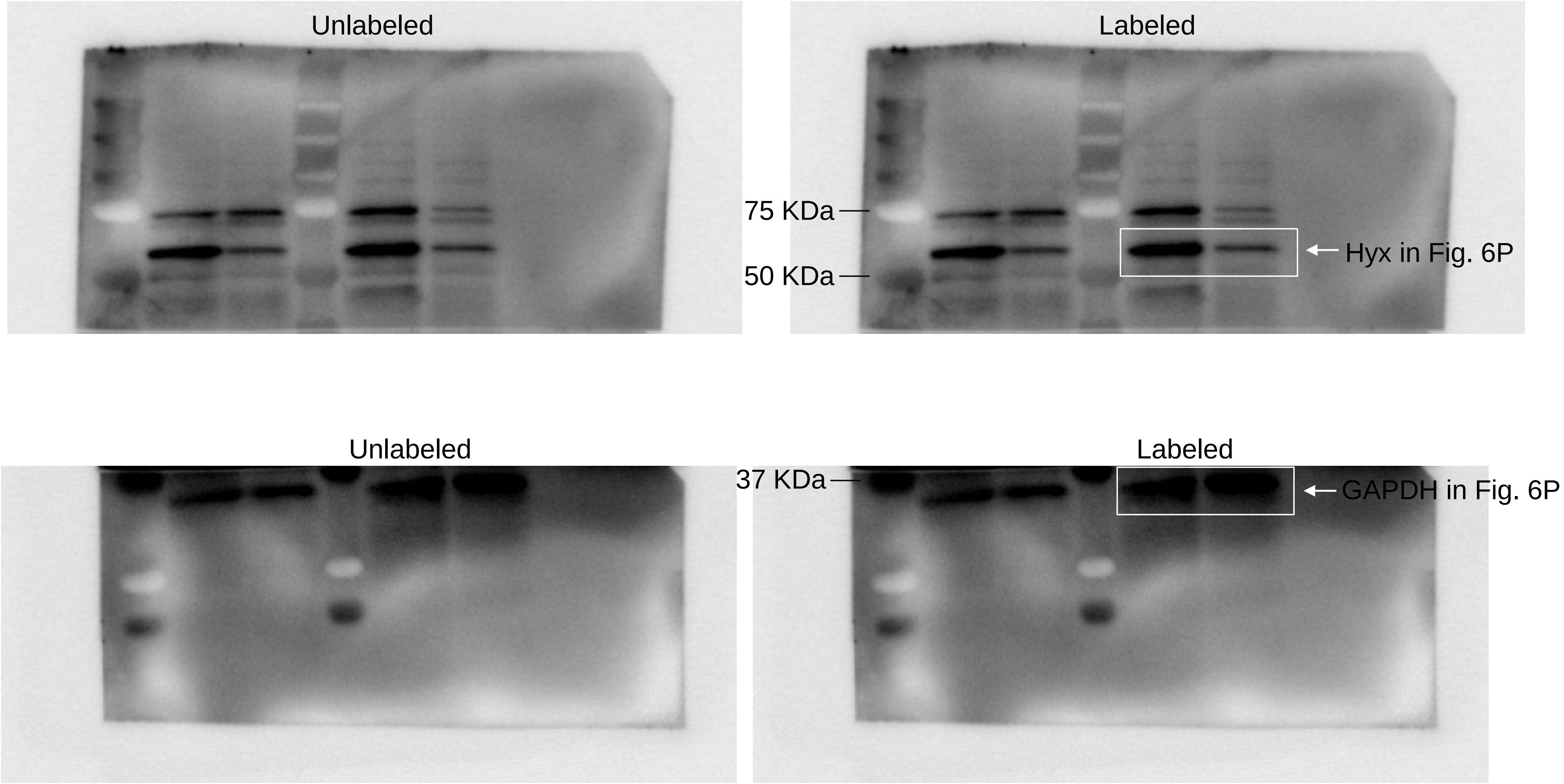

Original uncropped Western blot for Fig 6R

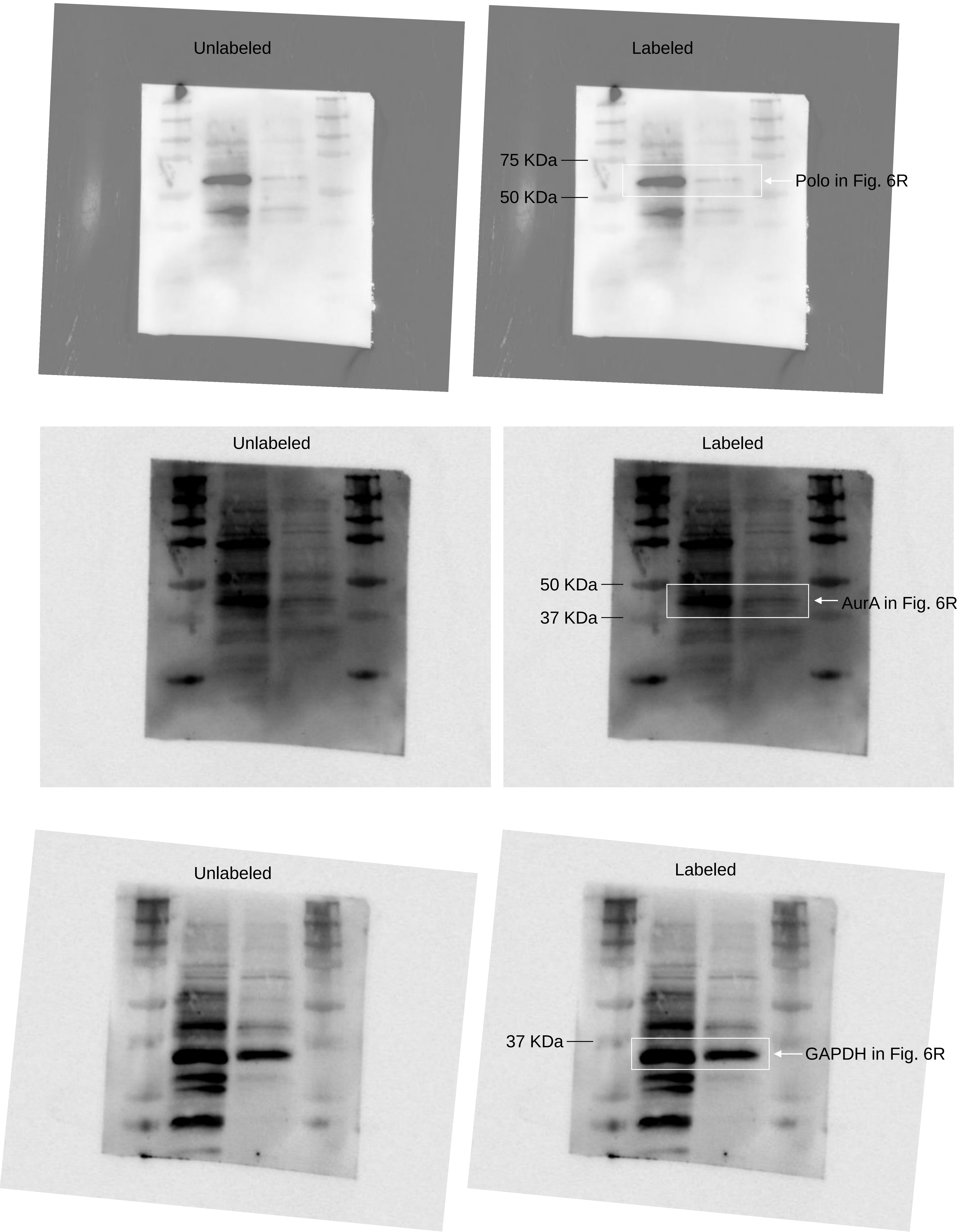

Supplement: S1 Raw Images — Hyx protein level is reduced in hyx loss of function larval brains. Western blotting analysis of 24 h AEL embryo extracts of control, hyxHT622, and hyxW12-46 as well as third instar larval brain extracts of control (UAS-β-gal Ri) and hyx Ri; hyxHT622/+ driven by insc-Gal4. Blots were probed with anti-Hyx antibody (upper panels) and anti-GAPDH antibody (lower panels). Both “Unlabeled” (left panels) and “Labeled” (right panels) uncropped original blots were provided. A protein ladder was indicated on the left of the “Labeled” membrane. Cropped images shown in S1D Fig were boxed and antibodies used were indicated by arrows. Original uncropped western blot for Fig 6P. Hyx is dramatically decreased upon hyx knockdown under the control of actin5C-Gal4. Western blotting analysis of larval brain protein extracts of control (UAS-β-Gal RNAi; UAS-β-Gal RNAi) and hyx knockdown with UAS-Dicer2 (hyx RNAi; UAS-Dicer2 RNAi) driven by actin5C-Gal4 at 48 h ALH. Blots were probed with anti-Hyx antibody (upper panels) and anti-GAPDH antibody (lower panels). Both “Unlabeled” (left panels) and “Labeled” (right panels) uncropped original blots were provided. A protein ladder was indicated on the left of the “Labeled” membrane. Cropped images used in Fig 6P were boxed and antibodies used were indicated by arrows. Original uncropped western blot for Fig 6R. Polo and AurA protein levels were significantly decreased upon hyx knockdown under the control of actin5C-Gal4. Western blotting analysis of 48 h ALH larval brain extracts of control (UAS-β-Gal RNAi; UAS-β-Gal RNAi) and hyx knockdown with UAS-Dicer2 (hyx RNAi; UAS-Dicer2 RNAi) under the control of actin5C-Gal4. Blots were probed with anti-Polo antibody (upper panels), anti-AurA antibody (middle panels), and anti-GAPDH antibody (lower panels). Both “Unlabeled” (left panels) and “Labeled” (right panels) uncropped original blots were provided. A protein ladder was indicated on the left of the “Labeled” membrane. Cropped images used in Fig 6 [file pbio.3001834.s014.pdf]
