# Supplementary material for: Parafibromin governs cell polarity and centrosome assembly in Drosophila neural stem cells
Source: PLoS Biol. 2022 Oct 12;20(10):e3001834. doi: 10.1371/journal.pbio.3001834 (PMC9555638; doi:10.1371/journal.pbio.3001834)
Supplement: Attachment — Submitted filename: Response letter for Hyx PLOS Biology HY 5pm 12 Sep 2022.pdf [file pbio.3001834.s018.pdf]

Point-to-point response to editor

We sincerely thank the Academic Editor for his/her insightful comments and have addressed all comments as the following.

1) Title: We think that the title should be edited slightly for brevity. If you agree, we would suggest changing it to "Parafibromin Governs Cell Polarity and Centrosome Assembly in Drosophila Neural Stem Cells"
Done.

2) Data: Thank you for providing the underlying data for each of your figures, as a supplementary file. Please reference this file in each of your figure legends (including supplemental). For example, you can add the statement, "the underlying data for this figure can be found in the supplementary file "Source Data".
Done.

3) Data: We require the original, uncropped and minimally adjusted images supporting all blot and gel results reported in an article's figures or Supporting Information files. We will require these files before a manuscript can be accepted so please prepare and upload them now. Please carefully read our guidelines for how to prepare and upload this data: https://journals.plos.org/plosbiology/s/figures#loc-blot-and-gel-reporting-requirements

>>Please provide the uncropped, minimally adjusted images relating to the blots presented in Figures 6P,R and Fig S1D
Done.

Summary:

- "Parafibromin can fully rescue ectopic NSCs and cell polarity defects in Drosophila hyx mutant brains" – although Parafibromin seems to rescue the ectopic NSCs these lineages are not completely wild-type like, this sentence should be changed as it does not fully rescue (even if the authors have not explored the observed lineage defects).

We have changed this sentence to "Human Parafibromin rescues ectopic NSC defects in *Drosophila hyx* mutant brains." in the abstract.

- "Hyx is required for the proper localization of a key centrosomal protein, Polo…" – I think the authors should also mention AurA.

We have changed it to "Hyx is required for the proper localization of two key centrosomal proteins, Polo and AurA." in the abstract.

- "overexpression of polo and aurA could significantly suppress ectopic NSC formation and NSC polarity defects" – the significance of these results is not shown in the figures as the authors have not performed a statistical analysis on these data (same in Significance section). Perform statistical analysis.

We have performed the statistical analysis and found that the ectopic NSC phenotype observed in *hyx* RNAi was significantly suppressed by *polo* or *aurA* overexpression in both type I and type II NSC lineages (Fig. 7C, E-F). The average type I NSC number per lineage in

hyx RNAi with *Venus-polo* and *aurA* overexpression was decreased to 1.7 ± 0.13 (n=66 NSC lineage) and 2.3 ± 0.32 (n=27 NSC lineage) (Fig. 7C; Mean ± SEM), significantly lower than 3.9 ± 0.77 (n=27 NSC lineage) in *hyx* RNAi with *β-gal* RNAi. Likewise, the average type II NSC number per lineage in *hyx* RNAi with *polo* and *aurA* overexpression was significantly dropped to 4.6 ± 0.40 (n=36 NSC lineage) and 4.5 ± 0.50 (n=21 NSC lineage), respectively, compared with 7.7 ± 1.4 (n=19 NSC lineage) in *hyx* RNAi with *β-gal* RNAi. Only one NSC per lineage was always observed in both type I and type II NSC lineages from *β-gal* RNAi control (n=20 for both type I and II NSC lineages), *Venus-polo* overexpression control (n=34 type I and 22 type II NSC lineage, respectively), and *aurA* overexpression control (n=36 type I and 24 type II NSC lineage, respectively).

We have also performed statistical analysis on NSC polarity defects and replaced previous Fig. 7 D-E with new quantification graphs in Fig. 7E-F, which indicated that the suppression of NSC polarity defects was statistically significant. We have included the above data on page 15-16 in the revised manuscript.

Results:

- (NSCs/clone) vs. (clones)– uniformize nomenclature.

Done. We have also indicated "All clones in the larval brains scored in this study were NSC clones." In the Materials and Methods section.

- "At 10hrs after pupa formation (APF), 88.2% of the NSCs in pupal brains exited the cell cycle…" – NSCs do not synchronously cycle through cell cycle and even in wild-type larval brains the rate of PH3 positive NSCs is much lower than <100%, and has been reported to be anywhere between 20%-50%. Furthermore, in wild type brains it has been shown that the majority of NSCs exit cell cycle ~24h APF. Thus, the percentage of PH3 positive cells does not reflect the number of NSCs that have not yet exited cell cycle, and an analysis at 10hAPF does not allow conclusions about NSC decommissioning. The authors must correct this paragraph and rethink their conclusion. As the time point analysed does not allow any conclusion about NSC cell cycle exit, the authors should include an alternative reason and discuss why they see an increase in PH3 positive NSCs in hyx mutants at 10h APF.

We have changed our statement to "increased number of *hyx*-depleted NSCs were still actively dividing in early pupal stages. This phenotype might be due to an NSC decommissioning defect or abnormal NSCs that are generated by symmetric divisions have gained proliferative potential." on page 6 of the revised manuscript.

- Fig S2C – missing wild-type Type II clone

We have included both wild-type Type I and Type II clones in Fig S2C.

- Fig S2C – Rescue by human HRPT2 does not seem to be complete: NB II seem to be smaller than WT NB II (although a control clone is missing); Lineage seems to be disorganized with increased number of Ase+Dpn+ cells and in ectopic locations – this should be discussed and the conclusion about full rescue toned down.

The smaller size of type II NB from *hyx* mutant with *HRPT2* rescue in previous Fig. S2C was due to a focal plane issue (which was not optimal for the NB). To avoid confusion of the readers, we have replaced images for the type II NB clone from *hyx* mutants with *HRPT2* overexpression with more representative images in Fig. S2C.

- Analysis of other components of the Paf1 complex – for the RNAis that did not cause a phenotype, the authors should reference manuscripts that confirm that these lines do knock down these genes effectively. The conclusion about the involvement of the Paf1 complex should be adjusted accordingly.

We have cited papers in the revised manuscript “Fly stocks and genetics” section on page 19 of the revised manuscript showing the effective knockdown of other Paf1 complex components, including Rtf1, Atu, Ctr9, and atms. At least one RNAi line per gene used in this study was shown to be effective by previous publications.

- “knocking down rtf resulted in a weak phenotype type II NSC overgrowth.. (rtf1 RNAi/BDSC#34586: type II, 31.2%, …” – clarify what overgrowth means, i.e. bigger NSCs, more NSCs? What do these percentages mean?

We have indicated that it meant more NSCs on page 7 of the revised manuscript. The percentage provided was the percentage of type II clones that displayed ectopic NSCs. We have indicated it in the Materials and Methods section on page 20.

- “…(Fig. 6I; 1.64-fold). Therefore, Hyx promotes the expression of both polo and aurA.” – In this section the authors have not yet shown that Hyx promotes the expression of these genes.

We have changed it to “Therefore, Hyx binds to the promoter region of both *polo* and *aurA*.”

- “By contrast, aPKC mRNA levels were increased to 5.21-fold (Fig S10K)” – This increase is non-significant and therefore this conclusion should be changed. aPKC's high variation should also be discussed.

We have changed the statement to “aPKC mRNA levels were not significantly affected (Fig S10K, 5.21-fold)”. The high variation of aPKC mRNA level might be due to unstable aPKC mRNA at the time point of analysis (48h ALH) when larvae started dying (page 14 of the revised manuscript).

- Ensure that the Gal4 lines used for each experiment are included in text or in the figure legend (eg. Fig 7A-B)

Done.

- Please check definition of all abbreviations (e.g. “BL” or RNAi).

We have defined all abbreviations when they first appeared in the manuscript text.

Discussion:

- Although the authors discuss the link between hyx and Polo and AurA they miss to include discussion about the polarity defects observed. I would recommend the authors including a couple of sentences about the polarity defects observed in hyx and also to reference previous literature to support their ideas.

We have included a brief discussion as “Mechanistically, Hyx promotes the expression of both *aurA* and *polo* by directly binding to the promoter region of these two genes. Hyx does not seem to affect the expression of a few other polarity genes we have tested, such as *apkc*, *baz*, *pins*, and *numb*.” on page 16.
