# Supplementary material for: Parafibromin governs cell polarity and centrosome assembly in Drosophila neural stem cells
Source: PLoS Biol. 2022 Oct 12;20(10):e3001834. doi: 10.1371/journal.pbio.3001834 (PMC9555638; doi:10.1371/journal.pbio.3001834)
Supplement: Attachment — Submitted filename: Response to reviewers Hyx PLOS Biology 4 May 2022 5pm.docx [file pbio.3001834.s015.docx]

**Point-to-point response to reviewers and Academic Editor**

**Reviewer #1**: Parafibromin is an evolutionarily conserved tumor suppressing gene which is known to underlie many cancers such as breast, gastric, colorectal, and lung cancer when lost. How defective Parafibromin leads to cancer initiation and progression remains poorly understood. The authors used fruit fly neural stem cells (neuroblasts) as a model system to characterize the functions of Parafibromin, Hyrax (Hyx), during asymmetric cell division By using a combination of genetic alleles and RNAi-transgenes, the authors described an avalanche of defects in cell polarity and centrosome spindle assembly leading to misspecification of cell identities and ectopic neuroblast formation. As an attempt to establish the mechanistic link between loss of hyx function and hyx-mutant phenotypes, the authors focused on aurA and polo, and provided data supporting a model that Hyx promotes AurA and Polo expression. The authors suggest that Parafibromin might suppress tumor formation these via similar mechanisms.

The authors did a marvelous amount of work to detail multiple aspects of mutant phenotypes in mitotic neuroblasts, making this study very exciting.

We sincerely thank the reviewer for the positive endorsement of our work.

However, it is unclear what the main cause for these phenotypes is, and the primary functions of Parafibromin are. For example, the authors show in Fig. 7 that Hyx may regulate the expression of polo via binding to the polo promotor, and suggest that mis-regulation of polo expression may be responsible for much of the Hyx mutant phenotypes. This is an excellent hypothesis to drill down a function of Hyx during asymmetric neuroblast division, as Polo kinase can regulate cell cycle, generation of microtubule asters, and protein polarization. However, the authors stopped short of providing any evidence that polo is a physiologically relevant target of Hyx in neuroblasts. The authors might be reluctant to test this hypothesis or already obtained inconclusive results as polarity defects in hyx-mutant neuroblasts appear to be much severer than the polo-mutant phenotypes that the same lab previously published (Wang et al., Nature 2007). If this is the case, could aurA be the target of Hyx as suggested by the authors? Again, polarity defects in hyx-mutant neuroblasts also appear to be much severer than the aurA-mutant phenotypes described in their previous study (Wang et al., G & D 2006). If neither polo nor aurA is a Hyx target, the data presented in this study are descriptive and cursive in regard to defining the true functions of Parafibromin.

We performed the luciferase assay to verify the direct binding of Hyx to the *polo* promoter. The endogenous Hyx in S2 cells was able to induce the luciferase reporter activity under the control of *polo*-promoter (poloPro; 643bp of sequences upstream of TSS) normalized against Renilla luciferase activity, but not with the vector control (Fig. 6J). We attempted to overexpress Hyx in S2 cells to test if it further enhances the luciferase activity under the control of the *polo* promoter. However, overexpression of Venus-tagged *hyx*-FL resulted in severe cell death (54.3%) detected by active Caspase-3 (Fig. S10 I-J; 11.2% cell death in the control), which precluded us from testing the effect of Hyx overexpression on the transcription of *polo* in the luciferase assay. Next, we sought to knock down *hyx* with dsRNA treatment in S2 cells and analyze the relative luciferase activity under the control of the *polo* promoter. The relative luciferase activity from the ds-*hyx* treatment group was significantly reduced to 0.5-fold compared with 1-fold from the control group (ds-*egfp*) (Fig. 6K) The relative luciferase activity driven by *actin5c* promoter induced by ds-*hyx* treatment and ds-*egfp* groups was indistinguishable (Fig. 6L; 1.0-fold *vs* 1.2-fold). We conclude that *hyx* can directly bind to the *polo* promoter region and promotes its transcription.

We have now performed genetic suppression experiments to show that both Polo and AurA are physiologically relevant targets of Hyx in NSCs. We overexpressed *polo* and *aurA* in the *hyx* RNAi knockdown background and found that the ectopic NSC phenotype caused by *hyx* depletion was significantly suppressed. With the introduction of *Venus*-*polo* and *aurA* into *hyx* RNAi, the average total NSC number of each brain was significantly reduced to 111.1±10.1 and 114.0± 5.1, respectively (Fig. 7B-C; n=7 BL and n=11 BL, respectively) compared with 133.8±4.0 NSCs in *hyx* RNAi alone (Fig. 7B-C; n=5 BL), close to 99.2±1.9 in control larval brains (Fig. 7B-C; n=5 BL).

Importantly, cell polarity defects caused by *hyx* depletion were also significantly suppressed by overexpression of *polo* and *aurA* in NSCs. The majority (72.7%) of *hyx* RNAi metaphase NSCs had lost aPKC polarity and the remaining 27.3% of NSCs had a weak aPKC crescent (Fig. 7D-E; n=55). Remarkably, 5.0% of *hyx* RNAi NSCs with *Venus-polo* overexpression formed a strong aPKC crescent and 47.5% showed a weak aPKC crescent (Fig. 7D-E; n=59 NSC). Similarly, a strong crescent was observed in 1.7% of *hyx* RNAi NSCs with *Venus-polo* overexpression and a weak Mira crescent was observed in 57.6% formed a weak Mira crescent (Fig. 7D, F; n=59), compared with 29.1% of NSCs with a weak Mira crescent in *hyx* knockdown alone (Fig. 7D, F; n=37). Likewise, *aurA* overexpression can dramatically restore the asymmetric localization of both aPKC and Mira in *hyx* RNAi NSCs. Upon *aurA* overexpression, a strong aPKC crescent was seen in 5.9% of *hyx* RNAi NSCs and a weak aPKC crescent was formed in 54.9% of NSCs (Fig. 7D-E; n=51 NSC). Similarly, upon *aurA* overexpression, 5.9% of *hyx* RNAi NSCs formed a strong crescent and 51.0% showed a weak Mira crescent (Fig. 7D, F; n=51 NSC). These suppressions were partial because the vast majority of control metaphase NSCs assembled strong aPKC and Mira crescent on the cortex (n=35).

We have now shown that Hyx also binds to the promoter region of *aurA*. ChIP-qPCR was performed along with a few other genes (as requested by other reviewers) including *polo*. After normalizing against “pre-serum” (1-fold), only a 1.37-fold increase was seen for the negative control. In contrast, 2.94-fold enrichment was observed for *orb2* promoter, a positive control. Consistent with our previous finding, Hyx binds to the promoter region of *polo* (new Fig. 6I; 2.63-fold and 2.95-fold using two pairs of primers). Hyx also binds to the promoter region of *aurA* (Fig. 6I; 2.85-fold), but not *numb* (Fig. 6I; 1.64-fold). Therefore, Hyx promotes the expression of both *polo* and *aurA*.

Note that previously we used purified rabbit IgG as a negative control for ChIP-qPCR experiments. In the new repeats of ChIP-qPCR experiments, we used pre-serum of the final bleed of anti-Hyx antibodies (used in ChIP) as the negative control, which is a better negative control than purified IgG. The fold change on polo promoter region was reduced when using “pre-serum” as the new IP control. We have replaced the previous ChIP-qPCR data with the new sets of data performed with pre-serum control in the revised manuscript (Fig. 6I).

We have included these new data in Figure 6I-L and Figure 7B-K and in the manuscript on Page 13-15.

There are many other important questions that the authors should clarify. Based on the number of cells in their clonal analyses, I would imagine that there must be significant cell cycle delay, which would tie into loss of Polo and AurA expression. Could Hyx be regulating other cell cycle regulators including PP2A, Cdc25, etc.?

We have examined cell cycle regulator String (Stg)/Cdc25 in *hyx* RNAi NSCs and found that its protein levels were unaffected as compared with that in control NSCs (Fig. S4C-D; 1.01-fold vs. 1.0-fold). Similarly, microtubule star (Mts), the PP2A catalytic subunit C, was unaffected in *hyx^HT622^* NSCs (Fig. S4E-F; intensity normalized against Dpn: 0.89 in control vs 0.78 in *hyx^HT622^*). We have included these new data in Figure S4 and in the manuscript on page 8.

In addition, cells including neuroblasts in hyx-mutant clones appear consistently smaller than cells in the control clones. Could Hyx be regulating genes in the PI3K pathway as the Wang lab also previously showed that this pathway plays a role in regulating asymmetric neuroblast division. The Wang lab has deep knowledge in neuroblast biology and asymmetric division, and a long history of publishing high-quality studies. I would expect that they can make more effort in drilling down the true cause of hyx phenotypes and defining the mechanisms by which this important tumor suppressor regulates cell proliferation.

We have examined the phosphorylated Akt (Ser505), an active form of Akt and commonly used as a readout of PI3K pathway activity. We found that the protein levels of P-Akt were unaffected in *hyx* RNAi NSCs as compared with that in control NSCs (Figure S4A-B; 1.0-fold vs. 1.02-fold), suggesting that Hyx does not seem to regulate PI3K/Akt pathway in NSCs. The smaller NSCs in *hyx* mutants likely result from the frequent symmetric division of *hyx* mutant NSCs that do not manage to grow back to their original size. We have included this new data in Fig. S4A-B and a short discussion on page 8 of the revised manuscript.

**Reviewer #2**: In this study, Deng and colleagues describe the phenotype of Parafibromin/Hyrax mutants in neural stem cells(NSCs). Using RNAi and clonal analyses of Hyx mutations, they show that that loss of Hyx triggers NCS amplification of type I and II NSCs. This phenotype is associated with a loss of the cortical apical PAR complex, loss of the spindle orientation machinery (Pins). Cortical proteins such as Miranda, Numb and Brat are also not targeted at the basal cortex. Overall this suggests loss of Polarity. This phenotype appears to be completely rescued by the expression of the Human Parafibromin, suggesting conservation of function between flies and humans.

Parafibromin is a member of the Paf complex. Neither the other members of this complex appear to produce ectopic neuroblasts suggesting Hyx mode of action is different of the Paf complex.

Additional defects are also detected in NSCs and S2 cells. Loss of centrosome maturation and nucleation during cell division, the presences of shorter spindles, the loss of the apical MTOC during interphase are shown. The centrioles are not affected but the recruitment of PCM proteins is strongly decreased (Aurora A, Msps, D-TACC). The Polo protein kinase is also missing from centrioles. The levels of the mRNAs encoding the corresponding proteins seem also slightly diminished. Finally they also showed using CHIP experiments that Hyx is found at elevated levels on the Polo promoter suggesting that the Hyx phenotypes are caused by a decreased Polo expression.

This article is convincing and the phenotypes are clearly described, although there is room for improvement (quantitation). Therefore, I am convinced that Hyx is a tumour suppressor gene.

We thank the reviewer for the positive endorsement of our work.

I think the paper would benefit from being shortened as there is a redundancy in the results and the multiplication of figures with the same general message is of little interest: It is important to have tested RNAi, mutants in different cell types but some of the results would benefit from being collected in one or 2 supplementary figures.

We have moved some of the redundant data into 2 supplementary figures (the original Fig 1C (*hyx* RNAi data) becomes a new Fig. S1F, the original Fig. 5A-C is now Fig. S6A-C, and the original Fig. 5D-F is now Fig. S7D-F).

I regret that the molecular mechanism is not established. It is suggested that Hyx is a transcription factor that regulates the expression of Polo but the data from this work show at most a correlation.

Major point.

1-The lack of molecular mechanism is my main concern. I understand why the authors try to link the Hyx phenotype to the loss of Polo. The two mutants produce indistinguishable phenotypes. Although it is a reasonable hypothesis to assert that Hyx controls Polo expression, the data presented here are not sufficiently documented.

We performed the luciferase assay to verify the direct binding of Hyx to the *polo* promoter. The endogenous Hyx in S2 cells was able to induce the luciferase reporter activity under the control *polo*-promoter (poloPro; 643bp of sequences upstream of TSS) normalized against Renilla luciferase activity, but not with the vector control (Fig 6J). We attempted to overexpress Hyx in S2 cells to test if it further enhances the luciferase activity under the control of the *polo* promoter. However, Venus-tagged *hyx*-FL resulted in severe cell death (54.3%) of S2 cells detected by active Caspase-3 (Fig. S10I-J; 11.2% cell death in the control), which precluded us from testing the effect of Hyx overexpression on the transcription of *polo* in the luciferase assay. Next, we sought to knock down *hyx* with dsRNA treatment in S2 cells and analyze the relative luciferase activity under the control of the *polo* promoter. The relative luciferase activity from ds-*hyx* treatment group was significantly reduced to 0.5-fold compared with 1-fold from the control group (ds-*egfp*) (Fig. 6K) The relative luciferase activity driven by the *actin5c* promoter induced by ds-*hyx* treatment and ds-*egfp* groups was indistinguishable (Fig. 6L; 1.0-fold *vs* 1.2-fold). We conclude that *hyx* can directly bind to the *polo* promoter region and promotes its transcription (manuscript page 14).

Hyx down regulation could also regulate the expression of other tumour suppressor genes.

We have now shown that Hyx also regulates AurA (manuscript page 14), but not Akt or Mts (manuscript page 8) in NSCs.

We have now shown that Hyx also binds to the promoter region of *aurA*. ChIP-qPCR was performed along with a few other genes including *polo*. After normalizing against “Pre-serum” (1-fold), only a 1.37-fold increase was seen for the negative control. In contrast, 2.94-fold enrichment was observed for *orb2* promoter, a positive control. Consistent with our previous finding, Hyx binds to the promoter region of *polo* (new Fig. 6I; 2.63-fold and 2.95-fold using two pairs of primers). Hyx also binds to the promoter region of *aurA* (Fig. 6I; 2.85-fold). Therefore, Hyx promotes the expression of both *polo* and *aurA*.

We have examined the phosphorylated Akt (Ser505), an active form of Akt and commonly used as a readout of PI3K pathway activity. We found that the protein levels of P-Akt were unaffected in *hyx* RNAi NSCs as compared with that in control NSCs (Fig. S4A-B; 1.0-fold vs. 1.02-fold), suggesting that Hyx does not seem to regulate PI3K/Akt pathway in NSCs. Similarly, microtubule star (Mts), the PP2A catalytic subunit C, was unaffected in *hyx^HT622^* NSCs (Fig. S4E-F; intensity normalized against Dpn: 0.89 in control vs 0.78 in *hyx^HT622^*).

2-Even if such a correlation with Polo was established, a strong functional link between the two genes would still have to be demonstrated. This is undoubtedly the direction they should be heading. Some of the tools available to the community would undoubtedly allow progress to be made in this area, which would provide the missing elements regarding the molecular pathway involved.

We have now performed genetic suppression experiments to show that both Polo and AurA are physiologically relevant targets of Hyx in NSCs (manuscript page 15). We overexpressed *polo* and *aurA* in the *hyx* RNAi knockdown background and found that the ectopic NSC phenotype caused by *hyx* depletion was significantly suppressed. With the introduction of *Venus*-*polo* and *aurA* into *hyx* RNAi, the average total NSC number of each brain was significantly reduced to 111.1±10.1 (n=7 BL) and 114.0± 5.1 (n=11 BL), respectively, compared with 133.8±4.0 NSCs in *hyx* RNAi alone (n=5 BL), close to 99.2±1.9 in control larval brains (n=5 BL) (Fig. 7B-C).

Importantly, cell polarity defects caused by *hyx* depletion were also significantly suppressed by overexpression of *polo* and *aurA* in NSCs. The majority (72.7%) of *hyx* RNAi metaphase NSCs had lost aPKC polarity and the remaining 27.3% of NSCs had a weak aPKC crescent (Fig. 7D-E; n=55). Remarkably, 5.0% of *hyx* RNAi NSCs with *Venus-polo* overexpression formed a strong aPKC crescent and 47.5% showed a weak aPKC crescent (Fig. 7D-E; n=59 NSC). Similarly, a strong crescent was observed in 1.7% of *hyx* RNAi NSCs with *Venus-polo* overexpression and a weak Mira crescent was observed in 57.6% formed a weak Mira crescent (Fig. 7D, F; n=59), compared with 29.1% of NSCs with a weak Mira crescent in *hyx* knockdown alone (Fig. 7D, F; n=37). Likewise, *aurA* overexpression can dramatically restore the asymmetric localization of both aPKC and Mira in *hyx* RNAi NSCs. Upon *aurA* overexpression, a strong aPKC crescent was seen in 5.9% of *hyx* RNAi NSCs and a weak aPKC crescent was formed in 54.9% of NSCs (Fig. 7D-E; n=51 NSC). Similarly, upon *aurA* overexpression, 5.9% of *hyx* RNAi NSCs formed a strong crescent and 51.0% showed a weak Mira crescent (Fig. 7D, F; n=51 NSC). These suppressions were partial because the vast majority of control metaphase NSCs assembled strong aPKC and Mira crescent on the cortex (n=35).

Other points that nevertheless need to be addressed.

1-Localization of Hyx. I would like to see images with a better resolution (accompanied with a Western Blot of the null alleles or RNAi treated cells). Is the protein only nuclear or also associated with mitotic structures? This is an interesting point, especially because the authors showed Hyx displays Paf1-independent functions and seems to be required for centrosome maturation.

This is also important to check the quality of the Ab since CHIP experiments are performed.

Hyx is localized mainly in the nucleus (0.74) and has a weaker intensity in the cytoplasm (0.26) (Fig S1A-B; total Hyx intensity as 1.0 in NSC), but not on mitotic structures (manuscript page 5).

Please note that we did not claim that *hyx^HT622^ hyx^w12-46^* are null alleles. Instead, they are strong loss-of-function alleles as we indicated in our initial manuscript (manuscript page 5). We performed western blot with protein extracts from FRT82B, *hyx^HT622^*, and *hyx^w12-46^* homozygous embryos, as *hyx* homozygous mutants are embryonic lethal and do not survive to larval stages. Relative protein intensity of Hyx at 24 h after egg laying (AEL) was reduced to 0.31-fold and 0.26-fold in both *hyx^HT622^* and *hyx^w12-46^*, respectively (Fig. S1D-E; control, 1-fold). Maternal Hyx might partially contribute to the detected Hyx proteins in these samples. Hyx levels were also significantly reduced in *hyx* RNAi in *hyx^HT622^*/+ background to 0.22-fold in 3^rd^ instar larval brains driven by NSC-specific driver *insc*-Gal4 (Fig. S1D-E). These western blot results are consistent with our immunofluorescence data that Hyx is dramatically diminished with weak signal in these *hyx* mutants at the 3^rd^ instar larval stage (Fig. S1A, C). The Western blot result is described on manuscript page 5.

The immunofluorescence and Western blot were performed using anti-Hyx antibodies generated in our lab, while the anti-Hyx antibodies used in ChIP experiments in this study were obtained from John T. Lis lab, which was used for ChIP previously (Adelman et al., Mol Cell Biol 2006).

Fig. S1A-E and in the manuscript on page 5.

2-The quantitation of ectopic NSCs is not optimal. How many NSCs are present in the clones or in RNAi cells? I believe the authors should be able to provide the data with the appropriate statistical tests.

We have now included the quantification graph to show the ectopic NSC number per clone in *hyx^HT622^* and *hyx^w12-46^* (Fig. 1D) as well as in *hyx* RNAi NSCs (Fig. S1H) and manuscript page 6.

Figure 1: What exactly do the percentages mean? A graph may help.

We have included the quantification graph showing the percentage of clones/lineages containing ectopic NSCs from *hyx^HT622^*, *hyx^w12-46^* (Fig. 1C), and *hyx* RNAi (Fig. S1G), as well as the percentage of PH3-positive NSCs in each brain after 10-hour pupal formation (Fig. 1F) and in the manuscript on page 6.

3-Are Hyx RNAi NSCs, can produce tumors that kill the host when transplanted in the abdomen of flies? Can we reverse this phenotype? This assay was developed by co-authors of this manuscript and should be feasible.

We transplanted allografts containing *hyx^HT622^* and *hyx^w12-46^* homozygous clones as well as *hyx* RNAi cells from larval brains to the abdomen of the female adult hosts. *mira^zz176^* brain tissue as a positive control induced tumor formation, while wild-type brain allograft as negative control did not. The transplantation of *hyx^HT622^* and *hyx^w12-46^* homozygous clones cannot grow obviously in the hosts and no dead hosts were found (n=29 host and n=30 host, respectively). Similar to *hyx* mutant, *hyx* RNAi allograft did not grow and no dead hosts were observed (0 out of 30 hosts). Taken together, hyx-depleted cells cannot produce tumors in allografts. Perhaps *hyx*-depleted cells are small in size and with altered cell fate, thus unable to expand in this tumor assay. As such, we have removed ‘Tumor suppressor” from the title of our manuscript.

This new data is included in the manuscript (page 8).

4-The authors mentioned centriole overduplication. How can they make the difference between centriole overduplication, centriole fragmentation or cells with centrioles 4 resulting from cytokinesis failure (polyploid cells)? Please comment or be more careful with data interpretation.

No giant, polyploidy cells were observed for *hyx*-depleted brains, suggesting there was a delay in cytokinesis, but not cytokinesis failure in these cells. We could not rule out the possibility of centriole overduplication or fragmentation, although centrioles apparently are intact in super-resolution imaging in *hyx*-depleted S2 cells (Fig. 6F). Cytokinesis delay in *hyx*-depleted NSCs was shown in Fig. S5I and Fig. 2J still images, in which at 34 min (“Cytokinesis”) two daughter cells are still connected by the spindle midzone. We have modified the statement on multiple centriole phenotypes on page 10.

5- « The new role for Hyx in regulating microtubule growth and asymmetric divisions in NSCs, proposed in this study, supports our previous finding that microtubules play an essential role in NSC polarity (47). » This sentence is misleading and the data published by the groups of Doe, Knoblich, Jan, Gonzalez have shown that NSC Polarity is established in a MT-dependent manner. Therefore I rather believe that some MAPs (Msps) or tubulin-interacting proteins of the Tubulin chaperone complex (Arl2) may control polarity in a MT-independent manner. Please amend or comment to be more accurate.

We have changed the statement to the following sentence including appropriate references on manuscript page 17.

The new role for Hyx in regulating microtubule growth and asymmetric divisions in NSCs, proposed in this study is consistent with the previous finding that NSC polarity is dependent on microtubules.

6-It should be stated that centrosomal protein such as Msps/D-TACC, Cnn, may not be well recruited due defective centrosome maturation and not a direct effect of Hyx on their expression.

We have included this statement on manuscript page 17.

7-Figure 2. Please measure cell size (with appropriate membrane marker) to quantify asymmetry level, with Statistical tests.

To examine the daughter cell size asymmetry, we measured the ratio of NSC to GMC diameter at telophase. Cells were outlined by a membrane marker CD8-GFP driven by *insc*-Gal4. The NSC-to-GMC diameter ratio in *hyx* RNAi telophase NSCs was significantly reduced to 1.2 (Fig. 2H-I; n=4) compared with 1.8 (n=11) in control telophase NSCs. This data indicates that *hyx* depletion disrupts daughter cell size asymmetry of the NSCs. We have included this data in Figure 2H-I and in the manuscript on page 7.

8-Figure 4. Please measure MT signal and provide appropriate Statistical tests on a graph for the MT regrowth assays.

We have quantified the microtubule intensity and included quantification graphs in Figure 4 B, D, and on manuscript page 9. In all time points (except for t=0), microtubule intensity in *hyx*-depleted NSCs was significantly reduced compared with the control (Fig. 4A-D). The method of microtubule intensity quantification is updated in Materials and Methods (page 21).

9-Figure 5. P20. I don't understand the meaning of -tub delocalization with the associated numbers: 6.9% in controls, 93.1% in the first mutant? Please clarify.

γ-tub is strongly localized to the centrosomes in the vast majority of control interphase and metaphase NSCs and weakly at the centrosomes in the remaining 3.8% of interphase and 6.9% of metaphase NSCs. There is no delocalization of γ-tub in control NSCs. We have modified Fig 5 legend (now Fig. S6A-B legend) to improve the clarity:

(A) Interphase NSCs of MARCM clones in control (FRT82B) and *hyx^HT622^* were labelled for γ-tub, Asl, GFP, and PH3. γ-tub delocalization: control, 0%, n=26; *hyx^HT622^*, 95.7%, n=23; *hyx^w12-46^*, 54.8%, n=42.

(B) Metaphase NSCs of MARCM clones in control (FRT82B) and *hyx^HT622^* were labelled for γ-tub, Asl, GFP, and PH3. γ-tub delocalization: control, 0%, n=29; *hyx^HT622^*, 93.1%, n=29; *hyx^w12-46^*, 70.8%, n=24.

10-The sd numbers are missing in many legends.

We have added sd numbers in figure legends of Figure 1, 5-6, S1, and S4-10.

11- RT-PCR experiments in the whole brain with the Insc promoter are not appropriate because GAL4 expression only occurs in the central brain. The results of gene expression in the whole brain (with optic lobes) are therefore difficult to interpret. What is the internal control for this experiment? Why not looking at genes required for NCS polarisation.

To achieve a more complete depletion of *hyx* in most larval brain cells including the optic lobe, we attempted to knock down *hyx* by RNAi using a more ubiquitous driver *tub-Gal4*. However, even with the presence of *tub*-Gal80ts, *hyx* RNAi under *tub*-Gal4 caused larval death before 24h ALH. Therefore, it is challenging for us to repeat RT-qPCR experiments for *hyx* RNAi under the control of *tub*-Gal4. Although *insc*-Gal4 is not the perfect driver for RT-qPCR experiments, our results already showed a significant decrease in a few key genes including *polo* and *aurA*. We also performed the ChIP experiment to confirm that Hyx binds to the promoter region of both *polo* and *aurA* (Fig. 6I and manuscript page 14).

Internal control (references genes) used for RT-qPCR were: *rp49/Rpl32* (*Ribosomal protein L32*), *Sdh* (*Succinate dehydrogenase*), and *Gapdh1* (*Glyceraldehyde 3 phosphate dehydrogenase 1*). We have included this information on Materials and Methods page 21.

To test the effect of Hyx on cell polarity genes, we performed RT-qPCR on third instar larval brains from *hyx* RNAi; *hyx^HT622/+^* under the control of *insc*-Gal4. Various cell polarity genes are also downregulated upon *hyx* depletion in the larval brain: *baz*, 0.79-fold; *par6*, 0.38-fold; *aPKC*, 0.64-fold; *insc*, 0.32-fold; *pins*, 0.44-fold; *mira*, 0.28-fold; *numb*, 0.56-fold; *brat*, 0.36-fold; *pon*, 0.33-fold (Fig. S10K). This data suggests that the expression of multiple cell polarity genes in NSCs may be affected by *hyx* depletion. However, the limitation of RT-qPCR experiments using larval brains is that interpretation can be complicated due to the heterogeneity and cell fate changes in the larval brain. To be cautious, we also performed RT-qPCR in S2 cell with *hyx* knockdown and found that some of the cell polarity genes are downregulated (*baz*, *par6*, *pins*, *numb*, *brat*, *pon*), but not others (*apkc*, *insc*, *mira*) (Figure below). Since RT-qPCR data from larval brains and S2 cells are not completely consistent, further investigations are required to confirm if Hyx could directly regulate the expression of these cell polarity genes. Since we have already proved that Polo and AurA are two important targets of Hyx in NSCs, we feel that further experiments on the potential direct regulation of cell polarity genes can be performed in a follow-up study.

This new data is included in Figure S10K and in the manuscript on page 15.

12- I disagree with "Parafibromin/Hyx has a tumour suppressor-like function and plays a critical role in the asymmetric division of NSCs, by regulating microtubule growth and centrosomal assembly in these cells ». The data shown here is that Hyx also maintains NSC polarity. So it could be contribution of both.

We have modified this statement to the following sentence on page 16 (discussion section) of the manuscript:

Parafibromin/Hyx plays a critical role in the asymmetric division of NSCs, by maintaining NSC polarity and regulating microtubule/centrosomal assembly in these cells.

13.Figure 7. I am not familiar with CHIP experiments but I suggest to control Hyx interaction with other promoters: Negative and positive controls should be shown and not only Polo.

We have included the negative control - the intergenic sequence at 5kb downstream of *numb* gene and the positive control targeting the potential *orb2* promoter region (Adelman et al., Mol Cell Biol 2006) in the ChIP-qPCR assay in Figure 6I and legend, and modified manuscript text on page 14.

**Reviewer #3**: Deng and Wang et al. identify Drosophila hyrax (hyx), orthologous to human Parafibromin, as a regulator of larval neural stem cell (NSC) proliferation. Overall, the authors present an impressive body of work providing foundational evidence hyx functions to limit NSC overproliferation with roles in microtubule dynamics, centrosome regulation, and NSC polarization. Several strengths are noted: the authors validate phenotypes using multiple hyx alleles and similarly compare NSC-autonomous effects using inscGAL4>RNAi; the analyses are fairly comprehensive, with numerous components analyzed for each given experiment; thoughtfully considered approaches, including MARCM clones, genetic rescue, and live imaging support their conclusions. Some weaknesses include: some quantification is lacking, how or indeed whether the described phenotypes fit together is not addressed, and overall mechanistic insights into precisely how hyx participates in so many processes is lacking.

Nevertheless, the authors present ample analysis and preliminary data to inform future discovery of mechanistic players. Overall, I support publication of a revised manuscript. Most of the points outlined below could be addressed through changes to the text.

We thank the reviewer for the positive endorsement of our work.

Major Point:

1. The authors present a body of work examining hyx phenotypes, yet how they fit within a model of Hyx function remains unclear. In particular, it is unclear if the authors propose hyx regulates one or multiple targets, which (if any) processes might be correlated, etc. Even within the centrosome section, the authors assay the distribution of numerous proteins, including upstream regulators Polo and Aur A, the loss of which might account for loss of the downstream effectors, yet this is not discussed. For this reason, interpretation of the data is at times sparse. A model fit into an expanded discussion may be helpful.

We have now performed genetic suppression experiments to show that both Polo and AurA are physiologically relevant targets of Hyx in NSCs (manuscript page 15). We overexpressed *polo* and *aurA* in the *hyx* RNAi knockdown background and found that the ectopic NSC phenotype caused by *hyx* depletion was significantly suppressed. With the introduction of *Venus*-*polo* and *aurA* into *hyx* RNAi, the average total NSC number of each brain was significantly reduced to 111.1±10.1 and 114.0± 5.1, respectively (Fig. 7B-C; n=7 BL and n=11 BL, respectively) compared with 133.8±4.0 NSCs in *hyx* RNAi alone (Fig. 7B-C; n=5 BL), close to 99.2±1.9 in control larval brains (Fig. 7B-C; n=5 BL).

Importantly, cell polarity defects caused by *hyx* depletion were also significantly suppressed by overexpression of *polo* and *aurA* in NSCs. The majority (72.7%) of *hyx* RNAi metaphase NSCs had lost aPKC polarity and the remaining 27.3% of NSCs had a weak aPKC crescent (Fig. 7E-F; n=55). Remarkably, 5.0% of *hyx* RNAi NSCs with *Venus-polo* overexpression formed a strong aPKC crescent and 47.5% showed a weak aPKC crescent (Fig. 7E-F; n=59 NSC). Similarly, a strong crescent was observed in 1.7% of *hyx* RNAi NSCs with *Venus-polo* overexpression and a weak Mira crescent was observed in 57.6% formed a weak Mira crescent (Fig. 7E, G; n=59), compared with 29.1% of NSCs with a weak Mira crescent in *hyx* knockdown alone (Fig. 7E, G; n=37). Likewise, *aurA* overexpression can dramatically restore the asymmetric localization of both aPKC and Mira in *hyx* RNAi NSCs. Upon *aurA* overexpression, a strong aPKC crescent was seen in 5.9% of *hyx* RNAi NSCs and a weak aPKC crescent was formed in 54.9% of NSCs (Fig. 7E-F; n=51 NSC). Similarly, upon *aurA* overexpression, 5.9% of *hyx* RNAi NSCs formed a strong crescent and 51.0% showed a weak These suppressions were partial because the vast majority of control metaphase NSCs assembled strong aPKC and Mira crescent on the cortex (n=35).

Our data support a model in which Hyx promotes the expression of *polo* and *aurA* in NSCs, and in turn, regulates cell polarity and centrosome/microtubule assembly (the model in Figure 7G and manuscript page 15).

2. Data analysis was robust for some experiments, but not for others.

a. Fig 2, distribution of several polarity proteins show "100% delocalization," yet no quantification is presented. How were these phenotypes scored? Were images blinded to prevent bias?

We quantified the localization of polarity proteins by scoring the percentage of proper localization and delocalization. The phenotypes were shown as the percentage of delocalization with the sample size (number of NSCs scored). In this case, the mutant samples were not blinded, rather, we have two postdocs to quantify the phenotypes independently and the quantifications were similar and combined together.

b. Fig 3E and Fig 4, reduced microtubule signals as described in the text and legend - how was this assessed? It is also unclear what is meant by "microtubule mass" within the legend. Do the authors refer to microtubule density, width, intensity, bundling? More detail how phenotypes were measured is needed.

We refer to microtubule intensity in these experiments. We have now quantified the microtubule intensity and included quantification graphs in Figures 3H and 4B, D. These quantification graphs support the microtubule abnormalities in *hyx*-depleted cells.

c. For all figures, the exact statistical test used should be reported in the corresponding figure legend.

We have included exact statistical tests in figure legends for Fig. 1-7, S1, S6-10.

Minor Point:

1. The title should include the word 'Drosophila' to indicate neural functions were solely examined in this system.

We have included the word “*Drosophila*” in the title.

2. The authors produced an N-term antibody to Hyx, but do not confirm whether the HT622 allele produces a truncated product or whether expression of either mutant form is reduced. Immunofluorescence of Hyx is reduced in the mutant backgrounds, indicating protein levels are reduced, yet fluorescence levels were not quantified. To further elucidate the nature of these alleles, semiquantitative immunoblotting is advised.

We have included a quantification graph on Hyx immunofluorescence intensity in Figure S1C, which showed that Hyx intensity was reduced to 0.15-fold (n=25 NSCs) and 0.32-fold (n=28 NSCs) in *hyx^HT622^* and *hyx^w12-46^* NSCs, respectively, compared with 1-fold in control NSCs (Fig. S1A, C; n=20 NSCs).

We performed western blot with protein extracts from FRT82B, *hyx^HT622^*, and *hyx^w12-46^* homozygous embryos, as *hyx* homozygous mutants are embryonic lethal and do not survive to larval stages. Relative protein intensity of Hyx normalized against GAPDH at 24 h after egg laying (AEL) was reduced to 0.31-fold and 0.26-fold in both *hyx^HT622^* and *hyx^w12-46^*, respectively (Fig. S1D-E; control, 1-fold). Maternal Hyx might partially contribute to the detected Hyx proteins in these samples. Hyx levels were also significantly reduced in *hyx* RNAi in *hyx^HT622^*/+ background to 0.22-fold in 3^rd^ instar larval brains driven by NSC-specific driver *insc*-Gal4 (Fig. S1D-E). This western blot result is consistent with our immunofluorescence data that Hyx is dramatically diminished with weak signal in these *hyx* mutants at the 3^rd^ instar stage (Fig. S1A, C).

The predicted truncated Hyx protein produced by *hyx^HT622^* can be potentially recognized by the anti-Hyx antibody, as the antigen area is still present in the truncation. We detected a putative Hyx truncation with the predicted size once (but not in the repeats) at 10-16 hours after egg laying (AEL) (Figure below, left panels; arrow), but not at 24 h AEL, suggesting that this truncated Hyx protein is inherently unstable. At 10-16 h AEL, relative Hyx protein levels normalized against GAPDH were only reduced to 0.46-fold and 0.47-fold in *hyx^HT622^* and *hyx^w12-46^* larval brains, respectively, compared with 1-fold in control (quantification graph below), likely due to maternal effect.

We have included the western blot data of *hyx^HT622^* and *hyx^w12-46^* at 24h AEL in Figure S1D-E and updated the manuscript on page 5.

3. Hyx is expressed within NSCs, but also throughout the larval brain. It was unexpected, therefore, insc-driven RNAi would reduce total hyx mRNA by ~80% from total larval brain preparations. Further, Hyx staining seems more intense in NSC nuclei, although this is neither measured nor discussed. The authors might expand on this a bit in the text.

The qRT-PCR experiments were performed using *insc*>*hyx* RNAi in *hyx^HT622^* heterozygous background, which helped to reduce *hyx* mRNA levels (manuscript page 15). Immunofluorescence also showed that Hyx levels in the entire NSC lineage are reduced compared with the control (Fig. S10B; manuscript page 13).

Hyx is localized mainly in the nucleus (0.74) and has a weaker intensity in the cytoplasm (0.26) (Fig. S1A-B; total Hyx intensity as 1.0 in NSC; manuscript page 5).

4. Would the authors add discussion why hyx deficient NSCs may continue to proliferate after pupal formation? Might a regulator of this process be altered by loss of Hyx?

Temporal transcription factors are known to schedule Prospero-dependent cell cycle exit of NSCs at the end of larval stages (Maurange et al., Cell 2008). The abnormal *hyx* deficient NSCs often undergo symmetric division, which might result in a disruption of temporal factors transition and, in turn, continued to proliferation after pupal formation (manuscript page 6).

5. The data examining the role of Hyx potentially regulating transcription seems underdeveloped. Data are presented showing modestly reduced RNA levels for several centrosomes genes.

We performed the luciferase assay to verify the direct binding of Hyx to the *polo* promoter. The endogenous Hyx in S2 cells was able to induce the luciferase reporter activity under the control *polo*-promoter (poloPro; 643bp of sequences upstream of TSS) normalized against Renilla luciferase activity, but not with the vector control (Fig. 6J). We attempted to overexpress Hyx in S2 cells to test if it further enhances the luciferase activity under the control of the *polo* promoter. However, overexpression of Venus-tagged *hyx*-FL resulted in severe cell death (54.3%) detected by active Caspase-3 (Fig. S10I-J; 11.2% cell death in the control), which precluded us from testing the effect of Hyx overexpression on the transcription of *polo* in the luciferase assay. Next, we sought to knock down *hyx* with dsRNA treatment in S2 cells and analyze the relative luciferase activity under the control of the *polo* promoter. The relative luciferase activity from the ds-*hyx* treatment group was significantly reduced to 0.5-fold compared with 1-fold from the control group (ds-*egfp*) (Fig. 6K) The relative luciferase activity driven by *actin5c* promoter induced by ds-*hyx* treatment and ds-*egfp* groups was indistinguishable (Fig. 6L; 1.0-fold *vs* 1.2-fold). We conclude that *hyx* can directly bind to the *polo* promoter region and promotes its transcription. These new data are shown on manuscript page 14.

Earlier in the results, the authors test RNAi for other components within the Paf1 complex, but only rtf showed an NSC-expansion phenotype. Does this preclude a role for Hyx in transcription or not? It would be helpful for the authors to add discussion about this.

We have included the following discussion on this point on manuscript page 17:

Among Paf1 complex components, only *rtf1* RNAi resulted in weak ectopic type II NSCs (but no ectopic type I NSCs). This finding suggests that the function of Hyx in asymmetric cell division of NSCs is largely independent of other components of the Paf1 complex. As our data support the role of Hyx in transcriptional regulation, it appears that Hyx alone without other Paf1 components might be sufficient to promote the target gene transcription. Nevertheless, we cannot rule out the possibility that other Paf1 components are involved in the asymmetric division to a much lesser extent or have redundant functions. Generating different combinations of double mutants or double knockdown might help to further probe the function of other Paf1 components in the asymmetric cell division.

6. The authors show qPCR for several PCM genes comparing controls versus hyx RNAi within NSCs. It is unfortunate the experiment could not be done in hyx mutants for technical reasons (hence the need for clones for the other experiments), as it is likely the reported defects underestimate the contribution of Hyx to their regulation and also account for the more profound phenotype observed for protein localization to centrosomes. The authors should note this in the text. I similarly wondered if any of the polarity genes were similarly reduced in hyx deficient cells by qPCR or western blot. Did they look? If not, perhaps the authors could add some speculation in the discussion.

We agree with the reviewer that our RT-qPCR results likely underestimate the contribution of Hyx to the regulation of gene expression due to various technical limitations. We have included the following statement on page 14 of the revised manuscript:

As *hyx^HT622^* and *hyx^w12-46^* mutants are embryonically lethal, we could not perform the RT-qPCR experiments using the larval brains of these alleles. The reported downregulation of gene expression using *hyx* RNAi likely underestimates the contribution of Hyx to this regulation.

To test the effect of Hyx on cell polarity genes, we performed RT-qPCR on third instar larval brains from *hyx* RNAi; *hyx^HT622/+^* under the control of *insc*-Gal4. Various cell polarity genes are also downregulated upon *hyx* depletion in the larval brain: *baz*, 0.79-fold; *par6*, 0.38-fold; *aPKC*, 0.64-fold; *insc*, 0.32-fold; *pins*, 0.44-fold; *mira*, 0.28-fold; *numb*, 0.56-fold; *brat*, 0.36-fold; *pon*, 0.33-fold (Fig. S10K). This data suggests that the expression of multiple cell polarity genes in NSCs may be affected by *hyx* depletion. However, the limitation of RT-qPCR experiments using larval brains is that interpretation can be complicated due to heterogeneity of cell types and cell fate changes in the larval brain. Further investigations are required to confirm if Hyx could directly regulate the expression of these cell polarity genes. Since we have already proved that Polo and AurA are two important targets of Hyx in NSCs, we feel that further experiments on the potential direct regulation of cell polarity genes can be performed in a follow-up study.

This new data is included in Figure S10K and in the manuscript on page 15.

7. On page 8, the authors speculate the observed supernumerary centrosomes may be attributed to cytokinesis errors. They state ~39% of hyx HT622 clones show cytokinesis defects; however, data supporting this claim is not presented. This is further confounded on p. 13 where the authors suggest Hyx blocks centriole "overduplication." There are several possible reasons why excess centrioles may be detected within an NSC. Did the authors observe failed cytokinesis in their live imaging experiments? Are hyx mutant NSCs and their nuclei bigger, consistent with such a defect? Barring data to support either claim, simply stating the underlying mechanism remains unknown would suffice.

The image of *hyx^HT622^* NSC with cytokinesis phenotype was shown in Fig. S3I (now Fig. S5I), but the figure was not mentioned in the initial manuscript by mistake. We have corrected the mistake on manuscript page 10. No giant, polyploidy cells were observed for *hyx*-depleted brains, suggesting there was a delay in cytokinesis, but not cytokinesis failure in these cells. In addition, in live imaging, *hyx*-depleted NSCs spent a longer time during cytokinesis than the control (movie stills ‘Cytokinesis” in Fig. 2J/ Video 1 and 2).

8. In FlyBase, Centrosomin protein is noted as "Cnn" (not CNN, page 8).

We have changed CNN to Cnn throughout the text in the revised manuscript.

9. The section on S2 cells examining PCM recruitment would logically fit better before presentation of the qPCR results.

The section on S2 cells on PCM recruitment (previously in Figure 7B-H) is now in Figure 6A-G, which comes before the qPCR results (now in Figure 7A).

10. The ChIP experiment was a nice addition, although it was unclear why only polo data were presented, especially given changes in Polo levels were among the most modest as detected by inscGAL4-qPCR. Presumably other genes were also found to be targets via ChIP? In the legend, orb2 is listed as a positive control, but there is no reference provided and the data are not shown.

We have now shown that Hyx also binds to the promoter regions of *aurA,* in addition to *polo* (Fig. 6I and manuscript page 14). ChIP-qPCR was performed along with *polo*. After normalizing against the new IP control “Pre-serum” (1-fold), only a 1.37-fold increase was seen for the negative control. In contrast, 2.94-fold enrichment was observed for *orb2* promoter, a positive control (Adelman et al., Mol Cell Biol 2006). Consistent with our previous finding, Hyx binds to the promoter region of *polo* (new Fig. 6I; 2.95-fold and 2.63-fold using two pairs of primers). Hyx also binds to the promoter region of *aurA* (2.85-fold), but not on *numb* (1.64-fold) (Fig. 6I). Therefore, Hyx regulates the expression of both *polo* and *aurA*.

11. In the methods, instead of "the type II driver," the authors should list the exact GAL4 line used to generate clones.

We have indicated the genotype of “the type II driver” as “*worniu* (*wor*)-Gal4, *ase*-Gal80^ts^ UAS-CD8-GFP” in the methods on page 20.

12. The authors might double-check the scale bars used in Fig 1. Likewise, would the authors please zoom in a bit more to the outlined clones? Even the largest clone in Fig 1 occupies a relatively small area in cropped images. Using a different color to outline the NSC within the clone would also help.

We have checked and corrected the scale bars in Figure 1.

We have now shown zoomed-in images of Fig. 1. Arrows indicate NSCs within the clones.

13. In Fig 2, panel B is much more zoomed in, as a result, the distribution of the polarity markers is easier to visualize. Would the authors please similarly zoom in to the NSCs in the rest of the figure?

We have shown the zoomed-in images in Figure 2A-G.

14. In Fig 3, spindle length is quantified, yet it remains unclear if the reduced spindle length is an indirect consequence of a smaller cell size, as the hyx deficient cells are smaller. If the measurements are normalized to cell volume or diameter, are they still statistically different?

We have normalized the spindle length to the cell diameter of NSCs and found the mitotic spindle length was still significantly shortened in *hyx-*depleted NSCs. The relative spindle length was significantly shortened to 0.74-fold in *hyx* RNAi NSCs (Fig. 3E; n=15 NSCs) compared with 0.82-fold in control NSCs (n=32 NSCs). Similarly, the ratio of spindle length to cell diameter was significantly reduced to 0.70-fold in both *hyx^HT622^* (Fig. 3F; n=13) NSCs and *hyx^w12-46^* NSCs (n=21 NSCs) compared with 0.79-fold in control NSCs (n=22 NSCs). These data have been included in Figure 3E-F and on manuscript page 8.

15. For all single-channel images, signals would be best detected in greyscale. This is particularly helpful for fine structures, like microtubules. The use of blue LUT on a black background is also discouraged.

We have made changes to the images as suggested (Fig. 1, 3, 4, S1-3, S5, S9, and S10).

16. Fig 7G, this centrosome looks like it might be on its side. It may be more effective to present an en face view, as with the control.

We have replaced the super-resolution image of ds-*hyx* in S2 cells with an en face view in Fig 7G (now Fig. 6G). In 50.4% of ds-*hyx* cells, either the inner ring of CNN and γ-tubulin is smaller than that in control cells, or no ring structure was observed (Fig. 6G; page 13).

17. Please define the white versus yellow arrows, as in Fig S8.

We have changed yellow arrows to white arrows, as both of them indicate NSCs. We have now included the following sentence in the figure legend (now Fig. S10 legend):

White arrows indicate NSCs (A-B) and centrosomes (C-G), respectively.

COMMENTS FROM THE ACADEMIC EDITOR

I think that the topic is quite interesting and that the experiments are well done, however several of the conclusions are probably overstated as in the end most of the results could be an indirect consequence of, for instance, transcriptional downregulation of Polo. Their phenotypic characterizations are well done and correct, but they could be better integrated experimentally.

We sincerely thank the Academic Editor for recognizing the strength of our work.

Below are some concrete issues:

- With the current experiments the authors cannot say that Hyx is a tumor suppressor gene, as the extra NBs formed are in very low number, meaning they do not expand in number exponentially. It looks like the defects in asymmetric cell division causes a transitory increase in NB number, but that these abnormal cells do not keep expanding, probably as they are so small and with incorrect fates. Compare for instance Brat RNAi or Pros RNAi tumor-brain sizes and NB numbers to Hyx RNAi in Fig1C.

We have now removed the statement on *Drosophila* Hyx is a brain tumor suppressor-like protein from the manuscript, as the allograft transplantation of *hyx^HT622^* (n=29 host) and *hyx^w12-46^* (n=30 host) homozygous clones and *hyx* RNAi (n=30 host) larval brain cells did not induce tumors. As rightly pointed out by the Academic Editor, likely these *hyx* deficient NSCs are small in size and with altered cell fate, therefore, unable to expand as known tumor suppressors such as Brat or Pros. See manuscript page 8.

- The authors find several defects in microtubule growth, centrosome maturation, polarity protein localization but do little comprehensive analysis of the mechanistic epistasis of these events, and in the end most of these defects could be caused for instance by lower transcription levels of polo (polo knock down is known to cause defects in centrosomes, and in numb and aPKC localization in NBs). This is however not experimentally analysed. Additionally, the authors, in my opinion, overstate that Hyx regulates MT growth, Hyx regulate polarity protein localization, Hyx regulates recruitment of several centrosome proteins, as all of these could be indirect. This is not made clear.

We have performed epistasis analyses and found that overexpression of *polo* or *aurA* can significantly suppress the ectopic NSC numbers and cell polarity defects observed in *hyx*-depleted larval brains. We have also performed the luciferase assay, which supports the regulation of Hyx on the *polo* expression (details described below). As both Polo and AurA have known roles in regulating cell polarity and centrosome/microtubule functions, the effect of Hyx on cell polarity and microtubule growth is likely mediated via Polo and AurA.

We have now performed genetic suppression experiments to show that both Polo and AurA are physiologically relevant targets of Hyx in NSCs. We overexpressed *polo* and *aurA* in the *hyx* RNAi knockdown background and found that the ectopic NSC phenotype caused by *hyx* depletion was significantly suppressed. With the introduction of *Venus*-*polo* and *aurA* into *hyx* RNAi, the average total NSC number of each brain was significantly reduced to 111.1±10.1 and 114.0± 5.1, respectively (Fig. 7B-C; n=7 BL and n=11 BL, respectively) compared with 133.8±4.0 NSCs in *hyx* RNAi alone (Fig. 7B-C; n=5 BL), close to 99.2±1.9 in control larval brains (Fig. 7B-C; n=5 BL).

Importantly, cell polarity defects caused by *hyx* depletion were also significantly suppressed by overexpression of *polo* and *aurA* in NSCs. The majority (72.7%) of *hyx* RNAi metaphase NSCs had lost aPKC polarity and the remaining 27.3% of NSCs had a weak aPKC crescent (Fig. 7E-F; n=55). Remarkably, 5.0% of *hyx* RNAi NSCs with *Venus-polo* overexpression formed a strong aPKC crescent and 47.5% showed a weak aPKC crescent (Fig. 7E-F; n=59 NSC). Similarly, a strong crescent was observed in 1.7% of *hyx* RNAi NSCs with *Venus-polo* overexpression and a weak Mira crescent was observed in 57.6% formed a weak Mira crescent (Fig. 7E, G; n=59), compared with 29.1% of NSCs with a weak Mira crescent in *hyx* knockdown alone (Fig. 7E, G; n=37). Likewise, *aurA* overexpression can dramatically restore the asymmetric localization of both aPKC and Mira in *hyx* RNAi NSCs. Upon *aurA* overexpression, a strong aPKC crescent was seen in 5.9% of *hyx* RNAi NSCs and a weak aPKC crescent was formed in 54.9% of NSCs (Fig. 7E-F; n=51 NSC). Similarly, upon *aurA* overexpression, 5.9% of *hyx* RNAi NSCs formed a strong crescent and 51.0% showed a weak These suppressions were partial because the vast majority of control metaphase NSCs assembled strong aPKC and Mira crescent on the cortex (n=35).

We have included the new data and discussion in the manuscript (page 15-17).

- Hyx affects transcription levels of multiple centrosome proteins. Is this specific? Or is it affecting globally transcription levels? Could it also affect transcription levels of polarity proteins and the defects observed are a result of lower levels of these fate determinants in general, or some in particular?

Hyx does not seem to affect global transcription levels. Although RT-qPCR results from *hyx*-depleted larval brains suggest that transcription levels of multiple centrosome proteins and cell polarity proteins (see the following paragraph) are decreased, this result may be influenced by cell fate alterations upon *hyx* loss-of-function. RT-qPCR results from S2 cells suggest that some, but not all of them are affected at the transcriptional level.

To test the effect of Hyx on cell polarity genes, we performed RT-qPCR on third instar larval brains from *hyx* RNAi; *hyx^HT622/+^* under the control of *insc*-Gal4. Various cell polarity genes are also downregulated upon *hyx* depletion in the larval brain: *baz*, 0.79-fold; *par6*, 0.38-fold; *aPKC*, 0.64-fold; *insc*, 0.32-fold; *pins*, 0.44-fold; *mira*, 0.28-fold; *numb*, 0.56-fold; *brat*, 0.36-fold; *pon*, 0.33-fold (Fig. S10K). This data suggests that the expression of multiple cell polarity genes in NSCs may be affected by *hyx* depletion. Our ChIP experiments suggest that Hyx binds to both *polo* and *aurA* promoter regions, but not *numb* promoter (Fig. 6I), suggesting that Hyx does not seem to directly regulate all cell polarity/cell fate determinant genes. Further investigations are required to confirm if Hyx could directly regulate the expression of these cell polarity genes. Since we have already proved that Polo and AurA are two important targets of Hyx in NSCs, we feel that further experiments on the potential direct regulation of cell polarity genes can be performed in a follow-up study.

We have included the new data and discussion in the manuscript (page 15).

- The authors say that ~40% of Hyx mutant clones have cytokinesis defects but do not discuss how this could affect the fate of the resulting NBs. As the asymmetric distribution of polarity proteins affects fate this needs to be addressed.

As loss of cell polarity is also observed in *hyx*-depleted NSCs without cytokinesis delay, cytokinesis delay is unlikely the primary cause of the defects in cell polarity in these NSCs but might exacerbate the protein mis-segregation and, in turn, cell fate changes following the NSC division. We have included a short discussion on manuscript page 10.

- To determine that the phenotype is not caused by de-differentiation of INPs the authors knock down hyx in INPs, however, the authors do not show that the knock down in INPs is efficient. As the authors have Hyx antibody so this should be easy to test.

Hyx protein was almost undetectable in the INP lineages from *hyx* RNAi larval brain (n=45 INP lineages), in contrast to abundant Hyx in control INP lineages (Fig. S3B; n=40 INP lineages), suggesting an efficient knockdown in INPs. We have included this data in Figure S2B and in the manuscript text on page 7.
