# Supplementary material for: Parafibromin governs cell polarity and centrosome assembly in Drosophila neural stem cells
Source: PLoS Biol. 2022 Oct 12;20(10):e3001834. doi: 10.1371/journal.pbio.3001834 (PMC9555638; doi:10.1371/journal.pbio.3001834)
Supplement: Attachment — Submitted filename: Hyx Response letter 2nd revision 2 Aug 2022 final.docx [file pbio.3001834.s017.docx]

**Point-to-point response to reviewers**

**Reviewer #1:** The big question that this study set out to investigate is the function of Parafibromin in proliferating cells and how mis-regulation of this gene contributes to tumor formation. In this revised study, Deng et al. made excellent attempt to address the reviewer's critique, and newly disclosed data clarified some questions raised in the previous review. However, these data also suggested that the model put forth in Fig. 7G is overly simplistic and perhaps highly biased. The authors provided plenty of data to suggest that Parafibromin plays a role in regulating asymmetric cell division, cortical cell polarity and the mitotic machinery. Yet, it remains extremely confusing as how Parafibromin regulates cell proliferation. This is largely due to their inability to distinguish direct vs. indirect effect of removing/knocking down Parafibromin function. The reviewer wants to emphasize that this is not due to lack of effort or vigor from the authors themselves, but perhaps, the complexity of biology itself is the reason. The data presented in this study provides a useful framework that others who are interested in Parafibromin can build on in their future work. Thus, the reviewer supports publication of this study after addressing two lines of inquiries outlined below.

We sincerely thank the reviewer for supporting the publication of our work.

1. The reviewer wants to air a deep concern that it is highly unlikely that the plethora of hyx-mutant phenotypes (spindle defects, polarity defects and ectopic neuroblast formation) and the severity of these phenotypes are the consequence of reduced aurA and polo transcription. The discrepancy between nearly complete suppression of ectopic neuroblast formation and minute restoration of cell polarity protein localization is difficult to overlook. This might be partly due to lack of 2 important control genotypes (overexpression of AurA or Polo). The reviewer would like to insist that the authors examine these two control genotypes under identical conditions and integrate the results in the appropriate bar graphs and in their data interpretation. Furthermore, the authors should revise the Y-axis (0-150 instead of 90-150) used in presenting neuroblast quantification in Fig. 7C. As is, the effect of overexpressing AurA or Polo is overly exaggerated.

We have now included these two important controls (overexpression of Polo and AurA under *insc*-Gal4) and found that they are indistinguishable from wild-type control (Fig.7A-B). Larval brains with Polo (*venus-polo)* and AurA overexpression had 99.2 ± 2.4 central larval brain NSCs (n=10 BL) and 98.9 ± 3.6 NSCs (n=12 BL) per brain lobe, respectively, similar to 98.5 ± 2.8 NSCs (n=11 BL) in control brains (Fig. 7B). In addition, aPKC and Mira are properly localized at prometa/metaphase NSCs upon overexpression of Polo or AurA (Fig. 7C-E). Strong aPKC crescent was observed in 95.1% (n=58 NSCs) in control, 96.0% (n=25 NSCs) in *Venus-polo*, and 95.8% (n=24 NSCs) in *UAS-aurA* during prometa/metaphase. Likewise, basal Mira crescent was seen in the vast majority of the prometa/metaphase NSCs upon *polo* or *aurA* overexpression (Fig. 7E; Strong Mira crescent: control, 95.1%, n=58 NSCs; *Venus-polo*, 96.0%, n=25 NSCs; *UAS-aurA*, 91.7%, n=24 NSCs). These results support our conclusion that both Polo and AurA are physiologically relevant targets of Hyx in NSCs. We have included these new data in new Figure 7A-E and in the manuscript on Page 15.

The Y-axis (90-150) was automatically generated in Prism. We have changed the Y-axis to 0-150 in Fig. 7C (now Fig. 7B).

2. The authors mentioned in the discussion that previous studies suggest a role for Parafibromin in regulating a myriad of cell cycle and cell proliferation genes including CyclinD1 and C-Myc. I would like to see that the authors further elaborate this line of reasoning by incorporating their own data in the discussion. The authors should acknowledge that cell polarity phenotypes observed in hyx-mutant neuroblasts do not resemble either aurA or polo-mutant neuroblasts previously described by the Wang lab. The fact that all known apical and basal proteins simultaneously become cytoplasmically localized in hyx-mutant mitotic neuroblasts strongly suggests that there is collective failure of cell cycle control in multiple aspects. Thus, it is logical to propose that Parafibromin functions as an upstream regulator of numerous cell cycle genes including aurora A, polo, CyclinD1 and C-Myc. The authors should revise Fig. 7G by including this more inclusive model as an alternative to their preferred model or simply replacing their preferred model altogether.

Previous studies on Parafibromin on Cyclin D1 and C-Myc were from mammalian cells, but not from *Drosophila* (see references below; also cited in our manuscript). In addition, there is no evidence that *Drosophila* cyclin D and Myc are involved in NSC polarity. Therefore, we prefer not to incorporate Cyclin D and Myc into our model.

Yang, Y.-J., et al., *The tumor suppressor, parafibromin, mediates histone H3 K9 methylation for cyclin D1 repression.* Nucleic acids research, 2010. **38**(2): p. 382-390.

Woodard, G.E., et al., *Parafibromin, product of the hyperparathyroidism-jaw tumor syndrome gene HRPT2, regulates cyclin D1/PRAD1 expression.* Oncogene, 2005. **24**(7): p. 1272-6.

Lin, L., et al., *The parafibromin tumor suppressor protein inhibits cell proliferation by repression of the c-myc proto-oncogene.* Proc Natl Acad Sci U S A, 2008. **105**(45): p. 17420-5.

**Reviewer #2:** The manuscript has greatly improved.

We thank the reviewer for the positive endorsement of our work.

Nevertheless, I still have some comments that need to be addressed before publication (see below).

However, I feel it would be necessary to perform rescue experiments of the Hyx polarity phenotype using active and inactive Polo kinases and not only using the WT kinase (Figure 7).

We have included additional controls and analyses to support the genetic suppression data. 1) Larval brains upon overexpression of Polo and AurA under *insc*-Gal4 are indistinguishable from wild-type control, as the number of NSCs per brain lobe and aPKC/Mira cortical polarity were normal in these brains (Fig 7A-E). 2) We have now shown that shorter spindle phenotype in *hyx* RNAi was also significantly suppressed by *polo* and *aurA* overexpression (Fig. 7F-G; control, 0.90 ± 0.05, n=18 NSCs; 0.82 ± 0.06, n=23 NSCs; 0.92 ± 0.06, n=22 NSCs; 0.91 ± 0.06, n=27 NSCs). We have included these new data in Figure 7 and in the manuscript on page 15.

With the additional supporting evidence, we feel that performing rescue experiments using active and inactive Polo kinases is not essential and is beyond the scope of a revision.

Western Blot against Polo and AurA using Hyx RNAi brains would also be nice.

We have examined Polo and AurA proteins in *hyx*-depleted brains with a ubiquitous driver *actin5C*-Gal4 at 48h ALH by western blot. Both Polo (0.25-fold) and AurA (0.43-fold) levels normalized against GAPDH levels were dramatically decreased following *hyx* depletion, compared with 1-fold in control (Fig 6R-S). This result strongly supports our conclusion that the expression of *polo* and *AurA* in the larval brain depends on Hyx function. The new data is included in Figure 6R-S and in the manuscript on page 14.

P2. Abstract: « We have also discovered that Hyx is required for the formation of interphase microtubule-organizing center and mitotic spindles in NSCs.». Spindles do form in Hyx mutants but they are not normal. Please amend.

We have modified it in the abstract as “We have also discovered that Hyx is required for the **proper** formation of interphase microtubule-organizing center and mitotic spindles in NSCs” on page 2.

P5. HyxHT622 is a truncated protein, the anti-Hyx antibody raised against the (1-176 aa) should reveal the 248 aa protein and not the full length protein. FigS1: Why is the truncated HyxHt622 mutant appears at the same size as the WT Hyx protein in Western Blots ? This is a bit suspect.

As *hyx^HT622^* cannot survive to larval stages, we performed Western blot using embryonic protein extracts. The full-length Hyx protein was detected in *hyx^HT622^* embryos, most likely due to maternal effect, as the amount of full-length Hyx was decreased at later embryonic stages (compare 24h after egg laying (AEL) at lower panels and 10-16h AEL at upper panels; Figure below; 24h AEL data are shown in Fig. S1D-E of the manuscript).

The predicted truncated Hyx protein produced by *hyx^HT622^* can be potentially recognized by the anti-Hyx antibody, as the antigen area is still present in the truncation. We detected a putative Hyx truncation with the predicted size once (but not in the repeats) at 10-16 hours AEL (Figure below, left upper panel; red arrow), but not at 24 h AEL, suggesting that this truncated Hyx protein is inherently unstable.

P8.« Perhaps hyx-depleted cells are small in size and with altered cell fate, thus unable to expand in this tumour assay ». I don't understand why this would be the case. There are examples of small fly brain tumor that exhibits small neuroblast-like cells with altered cell fate and asymmetric cell division defects. These tissues produce lethal tumours when injected in host flies.

Many small neuroblast-like cells can expand in the tumor assay, presumably because they can regrow to reach the larger cell size for cell division. We have modified the statement to “Perhaps *hyx*-depleted cells are small in size with altered cell fate and **have reduced cell growth**, thus unable to expand in this tumour assay.” on page 8.

P10. The cytokinesis delay or defect description is unclear. The description of the phenotype doesn't make sense. If the author claim there is no cytokinesis defect, the extra Asl dots could be due to overduplication or centrosome fragmentation.

If there is a cytokinesis defect, this should be visible for some of the dividing cells observed by live-microscopy (Figure 2J). I don't believe that speculation on cell fate in this paragraph is needed. It is suggested that Asterless extra dots are dots are due to cytokinesis defect but this is not shown in this paper. These extra dots are not visible in 3J and 5A in interphase, but visible in 6D and S5 in mitotic. Therefore Centriole fragmentation appears to occur in mitotic cells.

Thanks for pointing out that Asterless extra dots are only seen in mitotic NSCs, but not interphase NSCs. Indeed, we re-examined Asterless in NSCs from *hyx^HT622^* MARCM clones and found that multiple centrioles labelled by Asl were only seen at metaphase NSCs (Fig. S5D; 28.1%, n=32), but not at interphase NSCs (Fig. S5D; n=20). In contrast, two Asl-positive centrioles were always seen at both interphase and metaphase NSCs from control MARCM clones (Fig. S5D; n=21 and n=20, respectively). This observation suggests that centriole fragmentation might occur in *hyx*-depleted mitotic cells. We have included this new data in Figure S5D and in the manuscript and modified the statements on the multiple centriole phenotypes on page 10. As suggested by the reviewer, we have removed the statement “As loss of cell polarity is also observed in *hyx*-depleted NSCs without cytokinesis delay, cytokinesis delay is unlikely the primary cause of the defects in cell polarity in these NSCs but might exacerbate the protein mis-segregation and, in turn, cell fate changes following the NSC division.” from the revised manuscript on page 10.

P12.Please perform a Western Blot to state for decreased Polo and AurA protein levels. The proteins might be well expressed but not well located at centrosomes.

We have examined Polo and AurA proteins in *hyx*-depleted brains with a ubiquitous driver *actin5C*-Gal4 at 48h ALH by western blot. Both Polo (0.25-fold) and AurA (0.43-fold) levels normalized against GAPDH levels were dramatically decreased following *hyx* depletion, compared with 1-fold in control (Fig 6R-S). This result strongly supports our conclusion that the expression of *polo* and *AurA* in the larval brain depends on Hyx function. The new data is included in Figure 6R-S and in the manuscript on page 14.

P15 « The reported downregulation of gene expression using hyx RNAi likely underestimates the contribution of Hyx to this regulation». I believe this is too speculative and should be kept for discussion or be amended.

The above-mentioned statement was suggested by reviewer #3. Now we have knocked down *hyx* using *actin5C*-Gal4 in the larval brain, which resulted in a better knockdown of Hyx throughout the brain (refer to the response to the next question for details). Since we have replaced the previous qPCR data (under *insc*-Gal4) with this new data, we have removed the above-mentioned statement from the revised manuscript.

P16. « To test the effect of Hyx on cell polarity genes, we performed RT-qPCR on third instar larval brains from hyx RNAi; hyxHT622/+ under the control of insc-Gal4. » Why not using an ubiquitous driver such as Actin-GAL4 or Tubulin-GAL4 instead of Insc that is only expressed in the central brain?

Previously, we attempted *hyx* knockdown using a ubiquitous driver *tubulin*-Gal4. However, even with the presence of tub-Gal80ts, *hyx* RNAi under *tub*-Gal4 caused larval lethality before 24h ALH. Here, we have knocked down *hyx* in the larval brain by RNAi using another ubiquitous driver *actin-Gal4*. However, *hyx* RNAi under *actin*-Gal4 caused larval lethality after 48h ALH. Therefore, we performed both RT-qPCR and Western blot experiments for *hyx* RNAi (with *UAS-Dicer2*) under the control of *actin5C*-Gal4 at 48 h ALH. *hyx* mRNA levels were dramatically reduced to 0.23-fold upon *hyx* knockdown under *actin*-Gal4 (Fig. 6M). The overall Hyx throughout *hyx*-depleted larval brains was significantly reduced (fluorescence intensity 19.7 ± 8.99 a.u, n=8 BL), compared with that in control (41.5 ± 13.25, n=10 BL) (Fig. 6N-O). Particularly, Hyx levels were dramatically decreased in these *hyx*-depleted brains (20.4 ± 12.92, n=99 NSCs), compared with that in control (107.1 ± 29.18, n=96 NSCs) (Fig. 6N-O).

Remarkably, mRNA levels of *polo* and *aurA* were dramatically decreased to 0.33-fold and 0.24-fold, respectively, following *hyx* knockdown under *actin5C*-Gal4 (Fig. 6M). This effect was much stronger than our previous result using *insc*-Gal4 (*polo*, 0.65-fold; *aurA*, 0.73-fold), supporting our conclusion that Hyx promotes the expression of both *polo* and *aurA.* Various centrosomal genes were significantly downregulated upon *hyx* depletion in the larval brain (Fig. 6M; *γ-tub*, 0.59-fold; *cnn*, 0.68-fold; *tacc*, 0.45-fold), but not *msps* (0.86-fold). Among cell polarity genes, only *par6* was significantly decreased to 0.68-fold (Fig. S10K), and *baz* (0.84-fold), *pins* (1.04-fold), *insc* (1.07-fold) and *numb* (1.81-fold) were unaffected. By contrast, *aPKC* mRNA levels were increased to 5.21-fold (Fig S10K).

Our previous qPCR results leads to a different conclusion that most cell polarity genes were downregulated upon *hyx* knockdown. Given that the samples from *actin5C*-Gal4-driven *hyx* RNAi were from a much earlier stage (48h ALH) than that of previous qPCR data (96h ALH) using *insc*-Gal4, current qPCR data is more likely to show the immediate effect of *hyx* knockdown on cell polarity genes. Data from 96h ALH might be affected by cell fate changes in the larval brain at that stage. Taken together, our data suggest that Hyx appears to primarily regulate the transcription of *polo* and *aurA*. This new data is included in Figure 6M-S and Figure S10K and in the manuscript on page 14.

Moreover, the western blot result showed that Hyx protein levels in *hyx* RNAi brain under *actin5C*-Gal4 were dramatically reduced to 0.34-fold in contrast to 1-fold in control (Fig. 6P-Q), indicating an efficient *hyx* knockdown using *actin5C*-Gal4. We have examined Polo and AurA proteins in *hyx*-depleted brains with a ubiquitous driver *actin5C*-Gal4 at 48h ALH by western blot. Both Polo (0.25-fold) and AurA (0.43-fold) levels normalized against GAPDH levels were dramatically decreased following *hyx* depletion, compared with 1-fold in control (Fig 6R-S). This result strongly supports our conclusion that the expression of *polo* and *AurA* in the larval brain depends on Hyx function. The new data is included in Figure 6R-S and in the manuscript on page 14.

P17 and rebuttal letter : « We have changed the statement to the following sentence including appropriate references on manuscript page 17.

The new role for Hyx in regulating microtubule growth and asymmetric divisions in NSCs, proposed in this study is consistent with the previous finding that NSC polarity is dependent on microtubules. "

This is my mistake in the original review: I meant that polarization, as described by Knoblich, Jan, Gonzalez groups, is a MT-independent process. Therefore the effect of Arl2, Msps on polarity is likely MT independent. This is extremely important to revise this statement.

In our previous paper (Keng Chen et al, JCB 2016) on Arl2, we described the potential reasons for the discrepancy in the role of microtubules on NSC polarity in earlier work. Basically, the view that NSC polarity is MT-independent was mostly based on Colcemid treatment experiments, but not validated by analysis of mutants defective in microtubule function. It remains possible that Colcemid treatment does not depolymerize all microtubules in NSCs. In our Arl2 paper, we used mutant analysis to show that microtubules are important for NSC polarity. Therefore, we have changed our statement to “The new role for Hyx in regulating microtubule growth and asymmetric divisions in NSCs, proposed in this study is consistent with **our** previous finding that NSC polarity is dependent on microtubules.” on page 17 of the revised manuscript.

P17. «Therefore, the function of Hyx/Parafibromin in regulating microtubule growth and centrosomal assembly is likely a general paradigm in cell division regulation, which might be disrupted in cancer cells ». There is no effect shown on MT growth in this study, but defective centrosome assembly and centrosomal MT nucleation. Please clarify in the text. Otherwise, it is possible to monitor MT growth using labelled plus end binding proteins

We have modified the text by removing “microtubule growth” to “Therefore, the function of Hyx/Parafibromin in regulating centrosomal assembly is likely a general paradigm in cell division regulation, which might be disrupted in cancer cells.” on page 17.

Please start the Y axis at 0 (Fig 7C).

We have revised the Y-axis as 0-150 instead of 90-150 in Fig. 7C (now Fig. 7B).

Figure 7G. I was intrigued by the arrow in dotted line between Aurora A and Polo. Does this suggest that Polo is activated by Aurora to promote cell polarisation? I believe this link has been clearly shown in a recent 2022 study. If this is the case please cite the appropriate reference. If Aurora A activates Polo to promote polarization, partial loss of both kinase could be relevant to explain the polarisation defect in Hyx RNAi or mutant cells.

The arrow in the dotted line between Aura and Polo was based on the recent 2022 study. We have cited the paper “The spindle assembly checkpoint and the spatial activation of Polo kinase determine the duration of cell division and prevent tumor formation” on page 15 as “Polo can be directly activated by AurA in *Drosophila* mitotic NSCs [83].”.

Fig S8G and H. Why gamma Tubulin is still recruited in Hyx RNAi cells although Polo and aurA are strongly decreased on these structures? This is different to what is happening in neuroblasts (Fig S9). Please comment.

Unlike in dividing neuroblasts, in S2 cells, γ-tubulin is important for the nucleation of both centrosomal microtubules and non-centrosomal microtubules, i.e. chromatin-mediated microtubule assembly (Nicole M. Mahoney et al., Current Biology 2006). Therefore, in S2 cells, even with a reduction of Polo and AurA on the centrosomes, γ-tubulin might be recruited to the spindle poles in a centrosome-independent manner. We have included a brief discussion on this point on page 17 of the revised manuscript.

Fig S10. Several other Polarity genes are down regulated in addition to AurA and Polo. It is therefore not surprising that AurA and Polo OE cells do moderately rescue Hyx knock down. It should be stated more clearly that Hyx defective polarisation phenotype regulates might be caused by diminished expression of more polarity genes that AurA and Polo. The rescue effects are very weak and the classification of phenotypes in Figure 7 based on crescent intensity appears highly subjective following AurA and Polo over expression. I believe this could be caused by the fact that overexpressed Polo is not activated by upstream kinases. I am wondering why the active Polo kinase variant (available in flies as UAS-PoloT182D constructs and generated separately by the groups of Archambault and Conde) was not used in the assay. This would have probably led to less ambiguous results.

As shown by our latest qPCR data (for details, please refer to our earlier response to the same review), upon *hyx* depletion in the larval brain, *polo* and *aurA* were the most severely downregulated genes, while most polarity genes were not significantly downregulated. We have included additional controls and analyses to support the genetic suppression data. 1) Larval brains upon overexpression of Polo and AurA under *insc*-Gal4 are indistinguishable from wild-type control, as the number of NSCs per brain lobe and aPKC/Mira cortical polarity were normal in these brains (Fig 7A-E). 2) We have now shown that shorter spindle phenotype in *hyx* RNAi was also significantly suppressed by *polo* and *aurA* overexpression (Fig. 7F-G; radio of spindle length to cell diameter: control, 0.90 ± 0.05, n=18 NSCs; 0.82 ± 0.06, n=23 NSCs; 0.92 ± 0.06, n=22 NSCs; 0.91 ± 0.06, n=27 NSCs). With the additional supporting evidence, we feel that performing rescue experiments using active and inactive Polo kinases is not essential and is beyond the scope of a revision. We have included these new data in Figure 7A-G and in the manuscript on pages 14-15.

**Reviewer #3:**

Overall, the authors did a nice job addressing the revisions. Of note, they further test their model describing polo and aur A as downstream transcriptional targets of hyx through generation of transcriptional fusions and convincing genetic suppression assays. The revised manuscript also includes improved quantification, additional controls (e.g., ChIP), and expanded statistics and details to the methods, increasing the rigor of the work. Importantly, the authors responded to requests to temper certain conclusions and clarify the molecular framework proposed for Hyx function; specifically, they suggest aspects of PCM recruitment and errant NSC symmetric divisions arise as downstream consequences to down-regulated polo and aur A. Consequently, the manuscript includes increased mechanistic insight and rigor and is now suitable for publication.

We sincerely thank the reviewer for supporting the publication of our work.

**Response to the Editor**

Reviewer 2 has commented that it would be necessary to perform additional rescue experiments. While we agree that these experiments would strengthen the study, after discussion with the Academic Editor, we think that these may be beyond the scope of a revision and we would not strictly require this new data for publication in PLOS Biology.

We sincerely thank the Editor for the constructive comments and agree that performing additional rescue experiments is beyond the scope of this revision.
